# Association of progression-free or event-free survival with overall survival in diffuse large B-cell lymphoma after immunochemotherapy: a systematic review

**DOI:** 10.1038/s41375-020-0963-1

**Published:** 2020-07-10

**Authors:** Jie Zhu, Yong Yang, Jin Tao, Shu-Lian Wang, Bo Chen, Jian-Rong Dai, Chen Hu, Shu-Nan Qi, Ye-Xiong Li

**Affiliations:** 1 National Cancer Center/National Clinical Research Center for Cancer/Cancer Hospital, Chinese Academy of Medical Sciences and Peking Union Medical College, Collaborative Innovation Center for Cancer Medicine, Beijing, P.R. China; 2 Department of Radiation Oncology, Sichuan Cancer Hospital and Institute, Sichuan Cancer Center, School of Medicine, University of Electronic Science and Technology of China, Chengdu, P.R. China; 3 Institute of Basic Medical Sciences, Department of Human Anatomy, Histology and Embryology, Neuroscience Center, Chinese Academy of Medical Sciences, School of Basic Medicine, Peking Union Medical College, Beijing, P.R. China; 4 Division of Biostatistics and Bioinformatics, Sidney Kimmel Comprehensive Cancer Center, Johns Hopkins University School of Medicine, Baltimore, MD USA

**Keywords:** B-cell lymphoma, Prognosis

## Abstract

To investigate progression-free survival (PFS) and event-free survival (EFS) as early efficacy endpoints in diffuse large B-cell lymphoma (DLBCL), this systematic review included phase III randomized controlled trials (RCTs), phase II trials, and retrospective studies in newly diagnosed DLBCL receiving rituximab-containing chemotherapy through databases search up to 2019. Quality control was performed, where studies with high risk of bias were excluded. Prediction models were first established using the RCTs, and then externally validated in the phase II and retrospective populations. Trial-level surrogacy analysis was conducted by correlating the logarithmic (log) hazard ratio (HR) for PFS or EFS and log HR for OS. Correlation analysis at treatment arm-level was performed between 1-, 2-, 3-, and 5-year PFS or EFS rates and 5-year OS. The correlation was evaluated using the Pearson correlation coefficient *r* in weighted linear regression, with weight equal to patient size. Sensitivity analyses were performed to assess the consistency of predictive model by leaving one subgroup of trials out at a time. Twenty-six phase III RCTs, 4 phase II trials and 47 retrospective studies were included. In trial-level surrogacy, PFS (*r*, 0.772; 95% confidence interval [CI], 0.471–0.913) or EFS (*r*, 0.838; 95% CI, 0.625–0.938) were associated with OS. For rituximab immunochemotherapy treatment arms in RCTs, there was a linear correlation between 1 and 5-year PFS (*r*, 0.813–0.873) or EFS (*r*, 0.853–0.931) and 5-year OS. Sensitivity analysis demonstrated reasonable overall consistency. The correlation between PFS and OS was externally validated using independent phase II, and retrospective data (*r*, 0.795–0.897). We recommend PFS and EFS as earlier efficacy endpoints in patients with DLBCL primarily treated with rituximab-containing immunochemotherapy.

## Introduction

Diffuse large B-cell lymphoma (DLBCL) is the most common aggressive lymphoma subtype. Immunochemotherapy, mostly with rituximab plus cyclophosphamide, doxorubicin, vincristine, and prednisone (R-CHOP), has become the standard treatment over the past decade [1–4]. However, 15–40% of patients are refractory to initial immunochemotherapy, or relapse after complete response (CR). Such patients have poor outcomes, mainly depending on the risk group [5]. There is an urgent need to find more effective agents or regimens for high-risk patients in the immunochemotherapy era.

Overall survival (OS) is the gold-standard treatment endpoint in randomized controlled trials (RCTs). However, OS as the primary endpoint requires a large sample size and long follow-up time to observe the survival benefit, leading to high clinical development costs and delays in introducing novel drugs. When used as the primary endpoints in clinical trials, early efficacy endpoints such as progression-free survival (PFS) and event-free survival (EFS) may require a smaller sample size and shorter evaluation time than OS, and have been established in some malignancies [6–8]. Trial- and individual-level studies have demonstrated that 24-month PFS and EFS may be considered the early efficacy endpoints for OS in DLBCL [9–12]. However, these studies may not be comprehensive because they only included available 13 RCTs willing to disclose individual patient data and were based on a subset of all potentially eligible trials [1–4, 12–21]. The association of PFS or EFS with OS has not been specifically addressed at trial- or treatment arm-level in RCTs on patients treated with immunotherapy; furthermore, its association and predictive value have not been externally validated. We investigated PFS and EFS as efficacy endpoints in DLBCL in the rituximab era through literature-based analysis at both trial- and treatment arm-level. The correlation between PFS and OS was validated in independent cohort studies to confirm its significant role in guiding clinical practice.

## Methods

### Literature search and study selection

#### Inclusion and exclusion criteria

This study was exempted from review by the institutional review board because it used existing data and enrolled no human subjects. The eligibility criteria included phase III RCTs, phase II trials, and retrospective studies investigating the long-term survival of DLBCL patients who received first-line rituximab-containing immunochemotherapy. Studies were excluded if they met any of the following conditions: phase I trial; transformed or relapsed/refractory DLBCL; inadequate survival data; serology-positive for HIV, hepatitis B/C virus, or Epstein–Barr virus; sample size of <100 patients per arm; or patients with DLBCL consisting of <80% of the whole-sample size.

#### Literature search

Studies published before 31 December 2019, were included via a systematic literature search of MEDLINE, Embase, and PubMed using the keyword “DLBCL AND rituximab” and with the restriction to RCT, phase II trial, and retrospective study. Formal publications and meeting abstracts were included. Two authors (J.Z. and J.T.) conducted the literature search independently, and reviewed the results with a third author (S.N.Q.). When disagreement in study inclusion was met, J.Z., J.T and S.N.Q. carefully reviewed the potential eligible study again. Disagreements about study inclusion were resolved by consensus.

#### RCT inclusion and quality control

All potentially eligible RCTs were assessed for risk of bias in seven domains (random sequence generation, allocation concealment, blinding of participants and personnel, blinding of outcome assessment, incomplete outcome data, selective reporting, and other bias) using the Cochrane Collaboration tool. All information available in the assessment was acquired from formal publications, meeting abstracts, trial registry information on ClinicalTrials.gov (www.clinicaltrials.gov), and e-mail contact with trial designers. RCTs with high risk of bias in any domain were excluded.

**Fig. 1:**
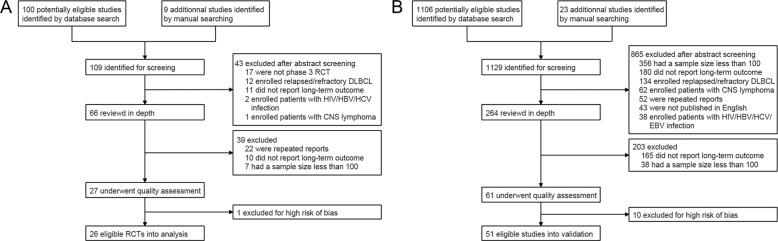
Flow chart for study inclusion. PRISMA flow charts for **a** phase III RCTs and **b** phase II and retrospective studies. RCTs randomized controlled trials.

**Fig. 2:**
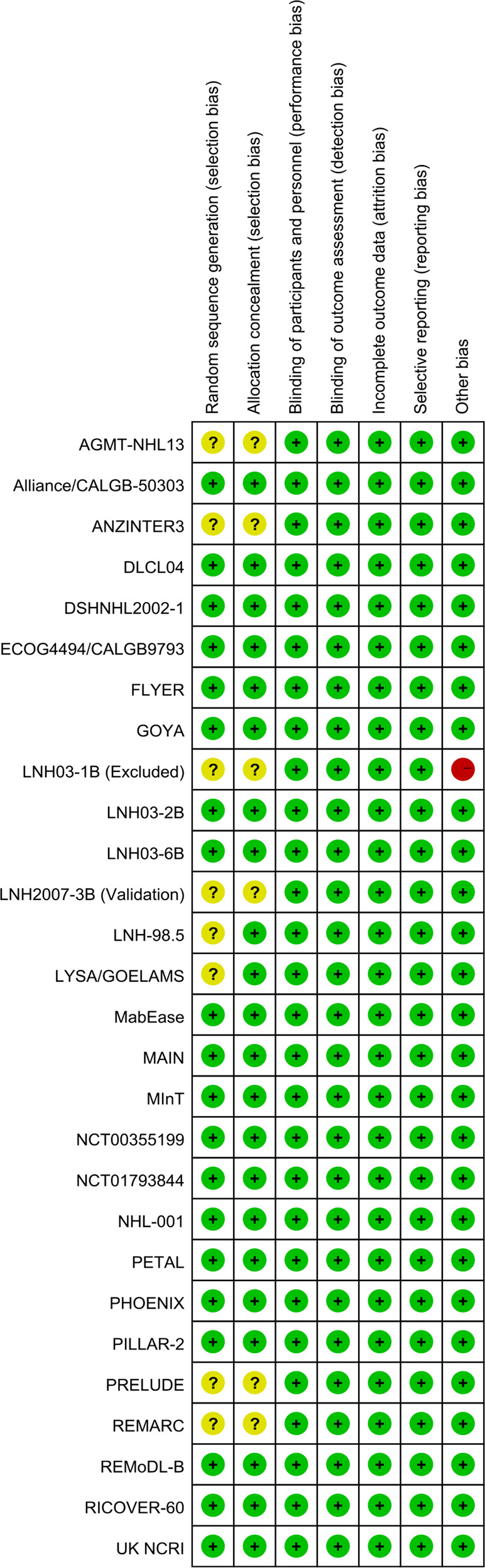
Summary of risk of bias in RCTs. “+” (green), “?” (yellow), and “−” (red) represent low, unclear, and high risk of bias, respectively. RCTs randomized controlled trials.

A total of 109 abstracts were reviewed. After excluding 43 ineligible records, the full texts of 66 records were reviewed. Thirty-nine unqualified records were excluded, and 27 RCTs were included in the quality assessment (Figs. 1a and 2; Supplemental Table 1) [1–4, 13–19, 22–37]. Seven trials were rated with unclear risk of selection bias because of the lack of comprehensive reporting on the randomization process. The LNH03-1B trial was excluded from the study because of the high risk of bias related to its premature close and a sample size far below statistical requirements (Fig. 2; Supplemental Table 1) [37]. Eventually, 26 qualified RCTs were included for trial- and treatment arm-level analyses (Table 1) [1–4, 13–19, 22–36]. According to the purposes of each trial, 26 RCTs were classified into 5 subgroups: (1) four RCTs (15%) compared R-CHOP (like) with CHOP (like) [1–4]; (2) ten (38%) RCTs compared R-CHOP (like) with rituximab+intensified/de-escalated chemotherapy [13, 15–17, 22, 23, 25–27, 31]; (3) nine (35%) investigated maintenance or consolidation therapy [3, 14, 18, 23, 24, 28–30, 32]; (4) three (12%) focused on R-CHOP+novel targeted therapy [19, 34, 35]; (5) two (8%) investigated the novel use of anti-CD20 monoclonal antibody [33, 36]. Of note, the two-stage randomized trial ECOG4494/CALGB9793 [3] and the 2 × 2 factorial randomized trial DLCL04 [23] were classified into 2 subgroups according to the respective research questions. These 26 RCTs included a total of 16,340 patients (median sample size, 623), with a median follow-up time of 2–10 years. The most common primary endpoints in these RCTs were EFS (*n* = 12, 46%) and PFS (*n* = 7, 27%), followed by disease free- or failure-free survival (*n* = 5, 20%), OS (*n* = 1, 4%), and CR (*n* = 1, 4%). The majority of RCTs (*n* = 20, 77%) used 2 or 3 years as the time point of the primary endpoint.

**Table 1:** Summary of phase III randomized controlled trials included in trial- and treatment arm-level analyses.

Trial	Inclusion Criteria	Primary endpoint	Median FU, year	No.	Treatment	PFS, %					EFS, %					OS, %	
						HR	1-y	2-y	3-y	5-y	HR	1-y	2-y	3-y	5-y	HR	5-y
*R-CHOP (like) vs. CHOP (like) (n* *=* *4)*																	
LNH-98.5 [1]	Age 60–80; stage II–IV; PS ≤2	3-y EFS	10	202	R-CHOP	0.61^P^	71.4	61	55.4	54*	0.6^P^	67.3	58.1	53	47*	0.69^P^	58*
				197	**CHOP**		54.1	40.8	35.1	30*		50.4	38.7	32.8	29*		45*
MInT [2]	Age 18–60; aaIPI ≤ 1; stage II–IV or bulky stage I	3-y EFS	6	413	R-CHOP-like	0.48*^P^	90.8	88.8	86.6	80.6	0.49*^P^	85	82	80.6	75.4	0.49*^P^	90.5
				411	**CHOP-like**		80.4	74.7	70.6	65.7		67.6	64.7	61.2	58.3		80.7
ECOG4494/CALGB9793 [3]	1st randomization: age ≥ 60; all stage; PS ≤ 3	3-y FFS	3.5	267	R-CHOP	NA	NA	NA	NA	NA	0.78*^P^ (FFS)	72.8	63.4	53*	46.8	0.83*^N^	58.4
				279	**CHOP**		NA	NA	NA	NA		63.2	51.4	46*	27.1		47.7
RICOVER-60 [4]	Age 61–80; all stages	3-y EFS	2.9	304	8 R-CHOP	0.67^P^	79.9	72.6	68.8*	62.8	0.64^P^	71.8	66.3	63.1*	58	0.84^N^	65.4
				306	6 R-CHOP	0.59^P^	83.8	76.9	73.4*	54	0.6^P^	75	69.2	66.5*	44	0.67^P^	64.4
				305	8 CHOP	0.96^N^	73.1	61.4	56.9*	50.4	0.81^N^	66.3	57.2	53*	47.2	1.02^N^	58.9
				307	**6 CHOP**		70.8	62.1	56.9*	43.1		59.3*	52.5	47.2*	35		57.7
*R-CHOP (like) vs. R* *+* *intensified/de-escalated chemotherapy (n* *=* *10)*																	
LNH03-2B [13]	Age 18–59; all stages; aaIPI 1	2-y EFS	3.7	196	R-ACVBP	0.48*^P^	91.6	89.7	87*	80.5	0.56*^P^	84.8	83.6	81*	74.6	0.44*^P^	91.6
				183	**R-CHOP**		81.8	74.6	73*	68.8		74.9	67.7	67*	62.5		79.9
ANZINTER3 [15]	Age > 65; stage II–IV; PS ≤ 3; “fit” in CGA	2-y EFS	3.5	114	R-miniCEOP	NA	NA	NA	NA	NA	1.12*^N^	63.3	54.4	47.7	46*	0.92*^N^	63*
				110	**R-CHOP**		NA	NA	NA	NA		64.4	56.7	52.7	48*		62*
LNH03-6B [16]	Age 60–80; aaIPI ≥ 1	2-y EFS	4.7	304	R-CHOP-14	0.99*^N^	75.9	62.8	60*	53.4*	1.04^N^	70.8	58.7	56*	50.3	0.96*^N^	65.8
				298	**R-CHOP-21**		77.4	66.2	62*	48.8*		75.4	64.6	60*	47.4		59.6
NCT01793844 [22]	Age ≥ 18; all stages; PS ≤ 3	3-y DFS	3.8	349	R-CHOP-14	1.10*^N^	74.7	65.8	63.2*	61.8	NA	NA	NA	NA	NA	0.98*^N^	74.4
				353	**R-CHOP-21**		78.5	70.2	66.1*	63.5		NA	NA	NA	NA		73.6
UK NCRI [17]	Age ≥ 18; stage IB-IV or bulky IA; PS ≤ 2	2-y OS	3.8	540	R-CHOP-14	0.94*^N^	83.7	74.7	72.8	66.7	NA	NA	NA	NA	NA	0.9*^N^	75.7
				540	**R-CHOP-21**		81.5	74.7	71.3	66.1		NA	NA	NA	NA		72.7
DLCL04 [23]	Age 18–65; DLBCL or FL 3b; stage II–IV; aaIPI 2–3; PS ≤ 2; high or intermediate-high risk	2-y FFS	6	196	R-MegaCHOP	NA	NA	NA	NA	NA	1.04*^N^ (FFS)	73.2	66*	64.1	62	1.14*^N^	76*
				203	**R-CHOP**		NA	NA	NA	NA		75.8	67*	64.2	64.1		79*
Alliance/CALGB 50303 [25]	Age ≥ 18; stage I (PMBCL) or II–IV; PS ≤ 2	PFS	5.2	241	DA-EPOCH-R	0.93*^N^	82.8	78.9*	75.8*	68*	NA	NA	NA	NA	NA	1.09*^N^	77.5*
				250	**R-CHOP**		80.7	75.5*	72*	66*		NA	NA	NA	NA		78.5*
FLYER [26]	Age 18–60; stage I–II; PS ≤ 1; tumor < 7.5 cm	3-y PFS	5.5	293	4 R-CHOP+2 R	0.91^N^	97.8	96.9	96*	94*	1.06^N^	91.0	89.7	89*	87*	0.85*^N^	97*
				295	**6 R-CHOP**		96.9	95.7	94*	94*		91.1	90.5	89*	88*		98*
PETAL [31]	Age 18–80; B- or T-cell; PS ≤ 3; PET (−)	2-y EFS	4.5	126	6 R-CHOP+2 R	NA	NA	77.5*	NA	NA	1.05*^N^	79.7	73.5*	70.1	61.8	0.88*^N^	77.9
				129	**6 R-CHOP**		NA	82*	NA	NA		82.3	76.4*	72.7	65.6		74
NHL-001 [27]	Age 16–60; DLBCL or FL 3b; all stages; PS ≤ 2	2-y PFS	3.8	134	R-CEOP90	0.44*^P^	91.5	88.8*	87.8	87.7	NA	NA	NA	NA	NA	0.80*^N^	89.9
				133	R-CEOP70	0.90*^N^	84.8	77.4*	76.3	76.3	NA	NA	NA	NA	NA	1.00*^N^	87.7
				133	**R-CHOP50**		84.6	75.9*	73.9	73.9		NA	NA	NA	NA		86.3
	Age 61–80; DLBCL or FL 3b stage I–IV; PS ≤ 2	2-y PFS	3.8	121	R-CEOP70	1.09*^N^	76.0	67.1	65.2	60.7	NA	NA	NA	NA	NA	1.02*^N^	68.5
				122	**R-CHOP50**		78.7	69.9	65.5	65.4		NA	NA	NA	NA		66.4
*R-CHOP (like) chemotherapy followed by maintenance/consolidation therapy (n* *=* *9)*																	
AGMT-NHL13 [18]	Age > 18; all stages; PS ≤ 2; CR/CRu	3-y EFS	3.8	338	R-CHOP-like+R maintenance	0.62*^P^	91.8	88.5	86.4*	82.8	0.79*^N^	88.9	83.7	80.1*	76.5	0.81*^N^	90.7
				345	**R-CHOP-like**		87.4	84.1	79*	68.8		88.8	81.5	76.5*	61.4		88.3
ECOG4494/CALGB9793 [3]	2nd randomization: age ≥ 60; all stage; PS ≤ 3; CR/PR	2-y FFS	3.5	174	R maintenance	NA	NA	NA	NA	NA	0.63*^P^ (FFS)	85.6	76*	63.8	NA	0.96*^N^	NA
				178	**Observation**		NA	NA	NA	NA		71	61*	56.7	52.7		NA
PRELUDE [28]	Age ≥ 18; stage bulky II or III or IV; IPI ≥ 3; PS ≤ 2; high risk; CR/CRu	3-y DFS	4	493	R-CHOP+enzastaurin maintenance	0.9*^N^ (DFS)	82.3*	78.1*	72.7*	NA	NA	NA	NA	NA	NA	1.04*^N^	76.4
				249	**R-CHOP**		82.8*	73.4*	69.6*	NA		NA	NA	NA	NA		75.6
REMARC [29]	Age 60–80; stage II–IV; aaIPI ≥1; PS ≤ 2; CR or PR	2-y PFS	3.3 (PFS), 4.3 (OS)	323	R-CHOP+lenalidomide maintenance	0.71*^P^	87.8	80*	74.4	61.4	NA	NA	NA	NA	NA	1.22*^N^	76.2
				327	**R-CHOP**		81.6	75*	67	49.3		NA	NA	NA	NA		77.7
PILLAR-2 [30]	Age > 18; stage II (tumor > 10 cm) or III or IV; IPI ≥ 3; CR	2-y DFS	4.2	372	R-chemo+everolimus maintenance	0.92*^N^ (DFS)	85.6	77.8*	71.9	65.2	NA	NA	NA	NA	NA	0.75*^N^	79.2
				370	**R-chemo**		80.6	77*	70.7	60.8		NA	NA	NA	NA		72.5
DSHNHL2002-1 [14]	Age < 61; stage III–IV; aaIPI 2–3; high risk	3-y EFS	3.5	132	R-MegaCHOEP+ASCT	1.16^N^	77	69.2	69.8*	61.3	1.3*^N^	68.1	61.6*	61.4	52.5	1.61^N^	71
				130	**R-CHOEP**		80	74.8	73.7*	68.5		74.6	70.9*	69.5	65.4		82.7
DLCL04 [23]	Age 18–65; DLBCL or FL 3b; stage II–IV; aaIPI 2–3; PS ≤ 2; high or intermediate-high risk	2-y FFS	6	199	R-HDC+ASCT	0.72*^N^	80.5	72*	70*	69.8	0.65*^P^ (FFS)	79.4	71*	70.5	69.4	0.98*^N^	78*
				200	**No ASCT**		70.6	65*	59*	54.8		70.2	62*	58.3	57.6		77*
NCT00355199 [24]	Age 18–65; stage III–IV or bulky II; high risk	3-y EFS	5	113	R-HDC+ASCT	0.84^N^	78.5	74.4	75*	74.2	0.99*^N^	66.9	65.2	65*	63.9	0.95*^N^	76.8
				122	**R-CHOP**		69.9	66.5	65*	62.9		68.4	62.4	62*	60.1		71.7
LYSA/GOELAMS [32]	Age: 18–75; stage I–II; tumor < 7 cm	5-y EFS	5.3	165	R-CHOP + RT	NA	NA	NA	NA	NA	0.61*^N^	98.6	97.3	95.7	92*	0.62*^N^	96*
				169	**R-CHOP**		NA	NA	NA	NA		96.5	93.3	91.4	89*		92*
*R-CHOP+novel targeted drug (n* *=* *3)*																	
MAIN [19]	Age ≥ 18; all stages	PFS to safety	2	390	RA-CHOP	1.09*^N^	75.1	68.3	60.8	NA	NA	NA	NA	NA	NA	1.03*^N^	NA
				397	**R-CHOP**		78.8	70.9	64	NA		NA	NA	NA	NA		NA
REMoDL-B [34]	Age ≥ 18; stage I (tumor > 10 cm) or II–IV; PS ≤ 2; with GEP	2.5-y PFS	2.5	358	RB-CHOP	0.86*^N^	81.1	76.1	75.3	68.1	NA	NA	NA	NA	NA	0.89*^N^	NA
				361	**R-CHOP**		78.4	71.9	70.2	65.6		NA	NA	NA	NA		NA
PHOENIX [35]	Age ≥ 18; non-GCB; stage II–IV; R-IPI ≥1; PS ≤ 2	EFS	2.9	419	R-CHOP+ibrutinib	0.92*^N^	NA	NA	70.8*	NA	0.93*^N^	81.3	73.3	69.6*	NA	0.99*^N^	NA
				419	**R-CHOP**		NA	NA	68.1*	NA		79.6	71.1	67.4*	NA		NA
*Anti-CD20 monoclonal antibody study (n* *=* *2)*																	
MabEase [36]	Age 18–80; tumor ≥ 1.5 cm; IPI 0 (≥7.5 cm) or 1–5; PS ≤ 2	CR/CRu	2.9	381	R(SC)+CHOP	1.3*^N^	85.2	75*	70	NA	1.18*^N^	77	68.6*	35.8	NA	1.3*^N^	NA
				195	**R(IV)+CHOP**		86.3	81.5*	75	NA		78.8	73.4*	67.3	NA		NA
GOYA [33]	Age ≥ 18; tumor > 1.5 cm; PS ≤ 2; IPI 0 (>7.5 cm) or 1 (age < 60) or ≥2; LVEF ≥ 50%	3-y PFS	2.4	706	G-CHOP	0.92*^N^	81.3	73	69.6*	NA	0.92*^N^	NA	NA	NA	NA	1*^N^	NA
				712	**R-CHOP**		79.9	70.8	66.9*	NA		NA	NA	NA	NA		NA

The standard arm is labeled in bold.

“P” and “N” in the top right of the HR indicate positive and negative results, respectively.

Trials: CALGB/Alliance 50303, Cancer and Leukemia Group B/Alliance 50303; DSHNHL2002-1, German High-Grade Non-Hodgkin Lymphoma Study Group 2002-1; ECOG4494/CALGB9793, The Eastern Cooperative Oncology Group 4494/Cancer and Leukemia Group B 9793; LNH98-5, Lymphome Non Hodgkinien study 98-5; LYSA/GOELAMS, Lymphoma Study Association/Groupe Ouest-Est d’études des Leucémies Aigües et autres Maladies du Sang; MInT, MabThera International Trial; PETAL, PET-Guided Therapy of Aggressive NHLs; RICOVER-60, rituximab with cyclophosphamide, doxorubicin, vincristine, and prednisone age > 60 years.

Chemotherapy regimens: CEOP, cyclophosphamide, epirubicin, vinblastine, and prednisone; CHOEP, cyclophosphamide, doxorubicin, vincristine, etoposide, and prednisone; CHOP, cyclophosphamide, doxorubicin, vincristine, and prednisone; DA-EPOCH-R, dose-adjusted etoposide, prednisone, vincristine sulfate, doxorubicin hydrochloride, cyclophosphamide, and rituximab; G-CHOP, obinutuzumab, cyclophosphamide, doxorubicin, vincristine, and prednisone; R, rituximab; R-ACVBP, rituximab, doxorubicin, cyclophosphamide, vindesine, bleomycin, and prednisone; R-chemo, rituximab-based chemotherapy; R-CEOP70: rituximab, cyclophosphamide, epirubicin (70 mg/m2), vincristine, and prednisone; R-CEOP90, rituximab, cyclophosphamide, epirubicin (90 mg/m2), vincristine, and prednisone; R-CHOEP, rituximab, cyclophosphamide, doxorubicin, vincristine, etoposide, and prednisone; R-CHOP, rituximab, cyclophosphamide, doxorubicin, vincristine, and prednisone; R-CHOP-14, R-CHOP every 14 days; R-CHOP-21, R-CHOP every 21 days; R-CHOP50, rituximab, cyclophosphamide, doxorubicin (50 mg/m2), vincristine, and prednisone; R-HDC, rituximab and high-dose chemotherapy; R-MegaCHOEP, R-CHOEP with escalated doses of cyclophosphamide, etoposide, and doxorubicin; R-MegaCHOP, R-CHOP with higher-dose cyclophosphamide and doxorubicin; R-miniCEOP, rituximab, cyclophosphamide, epirubicin, vinblastine, and prednisone; RA-CHOP, R-CHOP with bevacizumab; RB-CHOP, R-CHOP with bortezomib.

*aaIPI* age-adjusted International Prognostic Index, *ASCT* autologous stem cell transplantation, *CGA* comprehensive geriatric assessment, *CR* complete response, *CRu* unconfirmed CR, *DFS* disease-free survival, *DLBCL* diffuse large B-cell lymphoma, *EFS* event-free survival, *FFS* failure-free survival, *FL* follicular lymphoma, *FU* follow-up, *GCB* germinal center B-cell–like, *GEP* gene expression profiling, *HR* hazard ratio, *IPI* International Prognostic Index, *IV* intravenous, *LVEF* left ventricular ejection fraction, *NA* not available, *NHL* non-Hodgkin lymphoma, *No.* number of patients, *OS* overall survival, *PET* positron emission tomography, *PFS* progression-free survival, *PMBCL* primary mediastinal large B-cell lymphoma, PR partial response, *PS* performance status, *R-IPI* revised International Prognostic Index, *RT* radiotherapy, *SC* subcutaneous.

*Represents data directly reported in the full text.

#### Phase II trial and retrospective study inclusion and quality control

To validate the RCT findings, we analyzed the relationship between PFS and OS using phase II and retrospective data. For single-arm phase II trials and retrospective cohort studies, quality was assessed, with a maximum 9-star score, using the Newcastle–Ottawa scale (NOS) in terms of selection, comparability, and outcome [38]. Studies with low to moderate risk of bias (≥6 stars) were included in the statistical analysis. For the LNH2007-3B randomized phase II trial [39], the risk of selection bias was assessed using the Cochrane Collaboration tool. A total of 1129 abstracts were reviewed. After excluding 865 unqualified records, the full texts of 264 records were reviewed. We excluded 203 ineligible studies, and included 61 studies in the quality assessment (Supplemental Table 2). After excluding 10 studies with high risk of bias, a total of 47 retrospective studies and 4 phase II trials with 67 rituximab immunochemotherapy treatment arms were included in the external validation (Fig. 1b) [39–89]. The average NOS score was 6.9 stars. A total of 14,936 patients were included, with each arm containing 100–1322 patients (median, 177). The median follow-up time was 1.2–7.2 years (Table 2).

**Table 2:** Summary of phase II and retrospective studies used for predictive model validation.

Study	NOS (Stars)	Eligibility			Median	PFS (%)				5-y OS (%)
			Treatment	No.	FU (Years)	1-y	2-y	3-y	5-y	
*Phase II trial (n* *=* *4)*										
LNH2007-3B [39]	NA	Age 18–59; aaIPI 2–3	R-ACVBP	109	3.8	83.7	80.3	76.6	75.2	84.8
			R-CHOP	102		80.9	76.3	74.3	74.4	80.3
DENSE-R-CHOP-14 [40]	9	Age 61–80	6 R-CHOP+6 R	124	4.3	81.7	73.3	67.0*	55.7	62.3
LNH2003-3 [41]	9	Age 18–60; aaIPI 2	R-ACVBP+ASCT	157	3.8	84.8	79.6	77.8	76.5	78.6
Niitsu N, et al. [42]	9	Age 15–60; stage II–IV	R-CyclOBEAP	101	3.5	95.2	79.7	76.3	76.0*	85.0*
*Retrospective study (n* *=* *47)*										
Go SI, et al. [43]	7	PNI ≥ 40	R-CHOP	159	5.8	82.3	73.9	70.7	65.9	69.8
Lee J, et al. [44]	7	GCB Non-GCB	R-chemo	120	1.2	82.6	80.1	75.8	70.0*	71.0*
			R-chemo	177		77.4	69.8	68.0	65.0*	70.0*
Morrison VA, et al. [45]	7	All stages	R-chemo	1322	1.9	79.6	68.3*	63.1	47.7	67.4
Yim SK, et al. [46]	8	PET/CT score 1–3	R-CHOP	171	4.7	87.6	83.3	77.9	72.6*	78.1*
Chen Y, et al. [47]	6	BM PET/CT (−)	R-CHOP	147	2.5	87.9	82.6	81.5*	77.0	88.4
Hosoda Y, et al. [48]	7	All stages	R-CHOP	182	3.7	74.2	70.5	66.0*	52.1	66.6
Kim SH, et al. [49]	7	AGR ≥ 1.22	R-CHOP	139	5.5	82.2	74.9	73.0	69.8	70.8
Li LY, et al. [50]	7	BCL2 (+)	R-CHOP	145	1.9	48.6	38.5	36.2	36.0	45.2
Li YW, et al. [51]	7	Uric acid < 6.4 mg/dL	R-CHOP or like	114	1.8	90.8	86.4	83.5	82.8	83.0
Matsumoto K, et al. [52]	7	All stages	R-CHOP	185	4.6	85.4	80.5	76.1*	72.0*	80.1*
Sun FF, et al. [53]	7	ICPS 0	R-CHOP	202	2.6	93.2	86.9	86.5*	81.8	91.8
		ICPS 1	R-CHOP	144		87.9	83.8	82.3*	78.2	87.0
		ICPS 3	R-CHOP	119		65.6	60.0	54.5*	49.1	58.8
Go SI, et al. [54]	7	Sarcopenia-L3	R-CHOP	141	4.9	78.9	70.4	69.8	64.9*	67.8*
Kanemasa Y, et al. [55]	7	B2MG ≥ 3.2 mg/L	R-CHOP or like	101	3.1	62.6	46.8	45.3*	35.7	41.2
		B2MG < 3.2 mg/L	R-CHOP or like	173		90.3	85.5	79.7*	73.5	84.3
Li J, et al. [56]	7	AA genotype of EP300 SNP rs20551	R-CHOP	192	5.3	80.0	71.5	69.2	68.6*	77.0*
Liu YL, et al. [57]	7	TP53 Arg72	R-CHOP	238	4.7	77.1	67.6	64.0	63.5*	74.9*
Park YH, et al. [58]	7	High ALI	R-CHOP	130	4.6	91.1	85.1	78.8	77.3*	80.2*
Song MK, et al. [59]	7	No tumor necrosis	R-CHOP	387	4.1	86.0	75.5	72.3	68.3*	74.3*
Tsuyama N, et al. [60]	6	MYC (−), BCL2 (−)	R-CHOP	179	NA	85.2	78.3	75.5	69.0	81.2
Alinari L, et al. [61]	7	CD5+	R-chemo	102	3.3	64.0	43.2	40.0*	40.0*	60.0*
Prochazka KT, et al. [62]	6	Uric acid ≥ 6.8 mg/dL	R-chemo	130	NA	71.2	58.3	54.8	44.0*	50.4*
		Uric acid < 6.8 mg/dL	R-chemo	399		79.7	70.6	66.9	59.6*	66.2*
Seo S, et al. [63]	7	B2M ≥ 2.5 mg/L	R-CHOP	290	4	59.0	49.2	44.6	41.0*	49.2*
		B2M < 2.5 mg/L	R-CHOP	543		88.8	85.2	83.0	76.1*	83.8*
Dabaja BS, et al. [64]	7	All stages	R-CHOP+RT	293	4.5	96.4	90.8	88.5	83*	91.0*
		All stages	R-CHOP	548		93.9	87.3	83.3	76.0*	83.0*
El-Galaly TC, et al. [65]	6	IPI 0–1	R-CHOP like	138	2.4	93.4	89.2	89.1	86.8	90.7
		IPI 2	R-CHOP like	116		86.1	80.9	73.0	60.8	70.2
Gong QX, et al. [66]	7	CD30 (−)	R-CHOP	112	2.9	69.9	55.5	52.2	48.2	60.6
Kumar A, et al. [67]	7	Stage I/II	R-CHOP±RT	261	4.7	NA	NA	NA	82.0*	93.2*
Melchardt T, et al. [68]	6	NCCN-IPI 2-3	R-CHOP or like	199	4.3	85.3	77.4	73.9*	68.8*	77.3*
		NCCN-IPI 4-5	R-CHOP or like	189		78.5	66.6	63.5*	52.2*	56.4*
Nakajima Y, et al. [69]	7	Stage I/II, supradiaphragm	R-CHOP	109	4.3	90.9	88.8	86.6	86.4	92.2
Dabaja BS, et al. [70]	7	PET/CT (−)	R-chemo	239	3	89.0	84.3	81.0	78.0*	82.0*
Mian M, et al. [71]	6	All stages	R-CHOP	218	3.3	70.7	62.8	55.3	44.9	71.5
		All stages	R-COMP	146	1.5	74.7	60.0	56.3	50.4	61.6
Castillo JJ, et al. [72]	8	Asian patients	R-CHOP	455	3	83.4	72.7	65.0	60.0*	66.0*
		Western patients	R-CHOP	257		80.5	69.2	65.0	55.0*	64.0*
Hashimoto Y, et al. [73]	6	sIL-2Rα < 1000 U/mL	R-CHOP	101	2.2	90.0	83.3	83.4	82.0	84.0
Kojima M, et al. [74]	7	All stages	R-chemo	100	4.2	78.0	66.3	62.0*	61.4	66.0*
Lu HJ, et al. [75]	6	Stage I-III	R-CHOP	232	3.3	60.9	58.4	57.8	57.3*	69.8*
Ozbalak M, et al. [76]	8	All stages	R-CHOP	258	3.3	NA	NA	70.0*	41.0*	74.0*
Shi Z, et al. [77]	7	Stage III/IV	R-CHOP	110	2.7	NA	NA	NA	50.5*	72.9*
Tomita N, et al. [78]	7	Stage II	R-CHOP	190	4.3	90.4	87.0	84.7	84.0*	90.0*
Castillo JJ, et al. [79]	6	GC type	R-CHOP	379	NA	86.2	79.6	75.9	67.4	68.5
		Non-GC type	R-CHOP	333		81.3	73.5	70.0	63.6	64.0
Huang HH, et al. [80]	6	Age: 15–60 y; IPI ≥ 2	R-CHOP	112	5	94.7	69.7	50.3	40.9*	56.7*
Li ZM, et al. [81]	6	LMR > 2.6	R-CHOP	280	NA	91.3	85.8	83.6	79.4	83.2
		LMR ≤ 2.6	R-CHOP	158		76.6	66.6	60.8	54.2	64.6
Li XY, et al. [82]	7	All stages	R-CHOP	197	7.2	91.4	85.7	83.6	72.5	76.2
Lin TL, et al. [83]	6	Age > 60	R-chemo	189	NA	59.4	49.0	45.4	41.6	42.0
Tomita N, et al. [84]	7	Revised IPI 1-2	R-CHOP	201	3.6	88.5	86.2	81.7	79.0*	89.0*
		Revised IPI 3-5	R-CHOP	117		73.6	60.9	58.1	56.0*	63.0*
Sehn LH, et al. [85]	7	no BM involvement	R-CHOP	670	3.4	82.4	76.4	73.0*	68.5	73.5
Bari A, et al. [86]	7	All stages	R-chemo	271	3.4	78.3	68.0	65.5	53.2	60.0
Ennishi D, et al. [87]	7	All stages	R-CHOP	221	2.7	80.5	76.3	73.0	72.9	77.8
Phan J, et al. [88]	7	All stages	R-CHOP+RT	142	3	99.6	95.5	94.9	82.0*	91.0*
		All stages	R-CHOP	327		93.5	85.1	77.4	59.0*	68.0*
Scandurra M, et al. [89]	6	Without del (8p23·1)	R-CHOP	144	1.9	87.7	75.6	64.8	60.0	83.2

*Represents data directly reported in the full text.

Chemotherapy regimens: R, rituximab; R-ACVBP, rituximab, doxorubicin, cyclophosphamide, vindesine, bleomycin, and prednisone; R-chemo, rituximab-based chemotherapy; R-CHOP, rituximab, cyclophosphamide, doxorubicin, vincristine, and prednisone; R-COMP, rituximab, cyclophosphamide, non-pegylated liposomal doxorubicin, vincristine, and prednisone; R-CyclOBEAP, rituximab, cyclophosphamide, vincristine, bleomycin, etoposide, doxorubicin, and prednisolone. *aaIPI* age-adjusted International Prognostic Index, *AGR* albumin globulin ratio, *ALI* advanced lung cancer inflammation index, *Arg72* arginine at codon 72, *ASCT* autologous stem cell transplantation, *B2MG* beta-2 microglobulin, *BM* bone marrow, *DM* diabetes mellitus, *FU* follow-up, *GC* germinal center, *GCB* germinal center B-cell, *ICPS* inflammation-based cumulative prognostic score, *IPI* International Prognostic Index, *LMR* lymphocyte-to-monocyte ratio, *NA* not available, *NCCN-IPI* National Comprehensive Cancer Network–IPI, *No.* number of patients, *NOS* Newcastle–Ottawa scale, *OS* overall survival, *PET/CT* positron emission tomography/computed tomography, *PFS* progression-free survival, *PNI* prognostic nutritional index, *RT* radiotherapy, *sIL-2Rα* soluble interleukin-2 receptor-α, *SNP* single-nucleotide polymorphism.

### Statistical methods

#### Endpoint definition

In the RCTs [1–4, 13–19, 22–36, 39], OS was defined as the time from randomization to death from any cause. EFS was defined heterogeneously, but generally from randomization to any treatment failure, including disease progression, death, and treatment discontinuity for any reason (e.g., adverse effects or withdrawal). PFS was generally measured from the time of randomization to disease progression, relapse, or death from any cause (Supplemental Table 3). In the retrospective studies [43–89], OS was generally defined as the time from diagnosis or treatment to death from any cause, and PFS from diagnosis or treatment to disease progression, relapse, or death from any cause (Supplemental Table 4).

#### Data extraction

In the RCTs, patient characteristics, sample size, follow-up period, primary endpoint, standard and treatment arms, hazard ratio (HR), absolute EFS/PFS rates (year 1, 2, 3, 5), and 5-year OS were extracted (Table 1). For a repeatedly reported RCT, we included the most recent result with the longest follow-up time. All results of the standard and treatment arms were based on the intention-to-treat population. For the phase II trials and retrospective studies, patient characteristics, sample size, median follow-up time, treatment, absolute PFS rates (year 1, 2, 3, 5) and 5-year OS were extracted (Table 2). As described previously [90], the HR or survival rates at the different time points was extracted from the full text (labeled “*”) or the Kaplan–Meier survival curve using Engauge Digitizer software.

#### Correlation evaluation

The correlation analyses of the RCTs, weighted by trial size, were performed at both trial- and rituximab immunochemotherapy arm-level, without inclusion of treatment arms using conventional CHOP (like) regimen in arm-level analysis. At trial-level, the correlation of log HR (PFS) or log HR (EFS) with log HR (OS) was estimated using the Pearson correlation coefficient *r* in weighted linear regression, with weight equal to trial sample size. At rituximab immunochemotherapy arm-level, the linear correlation between the 1-, 2-, 3-, and 5-year PFS or EFS rates and 5-year OS rate was also evaluated by the correlation coefficient *r*, with weight depending on the sample size of each treatment arm. A strong association was indicated when the value of *r* was close to 1, and the 95% confidence intervals (CIs) of *r* were obtained using the bootstrap method with 1000 replications.

#### Sensitivity analysis

Phase III RCTs were classified into five subgroups according to study purposes. To assess the consistency and robustness of the developed predictive model across different settings, sensitivity analyses were performed by leaving each subgroup of trials out at a time. The correlation coefficient *r* and its 95% CI in trial-level and treatment arm-level correlation were reported similarly.

#### External validation of RCT prediction model in phase II trials and retrospective studies

We validated our finding by applying the predictive linear regression models to the phase II and retrospective studies with adequate survival data. The predicted 5-year OS rate was calculated from the actual 1–5-year PFS rates in the phase II or retrospective studies using the established linear regression model from the RCTs. For example, the equation “5-year OS = α × 1-, 2-, 3-, or 5-year PFS + β” was derived from the RCTs. Using the reported 1–5-year PFS rate derived from the phase II and retrospective studies, we used these models to generate the predicted 5-year OS rates. The actual and predicted 5-year OS rates were plotted in scatter plots. Statistical analysis was performed in SPSS (version 21.0, IBM Inc.); data visualization was performed using the ggplot2 package in R software (version 3.3.2, R Foundation for Statistical Computing).

### Data sharing statement

For original data, please contact yexiong12@163.com.

## Results

### Trial-level correlation between treatment effects of PFS or EFS on OS in RCTs

**Fig. 3:**
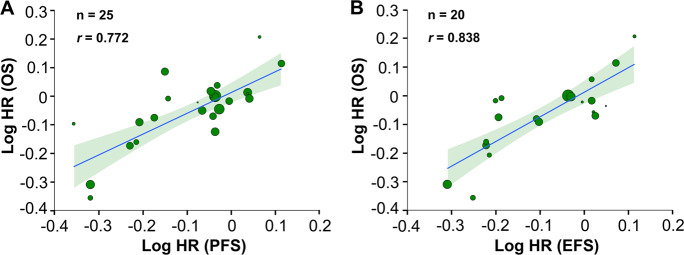
Trial-level Correlation Between Treatment Effects on PFS or EFS and OS in RCTs. Trial-level correlations between **a** HR for PFS and HR for OS, and **b** HR for EFS and HR for OS. Circle size is proportional to the number of patients in each comparison. The solid blue line indicates the fitted weighted linear regression line; the light green zone represents its 95% CI; *r* indicates the correlation coefficient. PFS progression-free survival; EFS event-free survival; OS overall survival; RCTs randomized controlled trials; HR hazard ratio; CI confidence interval.

Of 26 RCTs (Table 1), 20 (77%), 1 (4%), and 1 (4%) reported one, two, and three pairs of PFS HR and OS HR, respectively. A significant correlation was observed after analyzing 25 pairs of PFS HR and OS HR. Log HR (PFS) correlated with log HR (OS) (*r* = 0.772; 95% CI, 0.471–0.913; Fig. 3a). Sensitivity analyses showed good consistency in most subgroups, except when leaving the subgroup R-CHOP (like) vs. CHOP (like) out (*r* = 0.61; 95% CI, 0.075–0.863; Supplemental Fig. 1a). This result was expected. Among the 26 RCTs we studied, 4 trials [1, 2, 4, 13] were shown statistically significant OS benefits, including 3 trials [1, 2, 4] in the subgroup comparing R-CHOP (like) with CHOP (like). The exclusion of these positive trials at once naturally leads to a wider confidence interval.

Fourteen RCTs (54%) reported one pair of EFS HR and OS HR each (two treatment arms); three RCTs (12%) reported two pairs of EFS HR and OS HR each (four treatment arms). The analysis of 20 pairs of EFS HR and OS HR demonstrated that log HR (EFS) correlated with log HR (OS) (*r* = 0.838; 95% CI, 0.625–0.938; Fig. 3b). Sensitivity analyses demonstrated good consistency in most subgroups, except when leaving the subgroup R-CHOP (like) vs. CHOP (like) out (*r* = 0.732; 95% CI, 0.278–0.941) because of similar reasons as in PFS (Supplemental Fig. 1b). These results confirm that treatment gain in PFS or EFS can predict OS benefit at trial level with an acceptable consistency.

### Treatment arm-level correlation between PFS or EFS and OS in RCTs

**Fig. 4:**
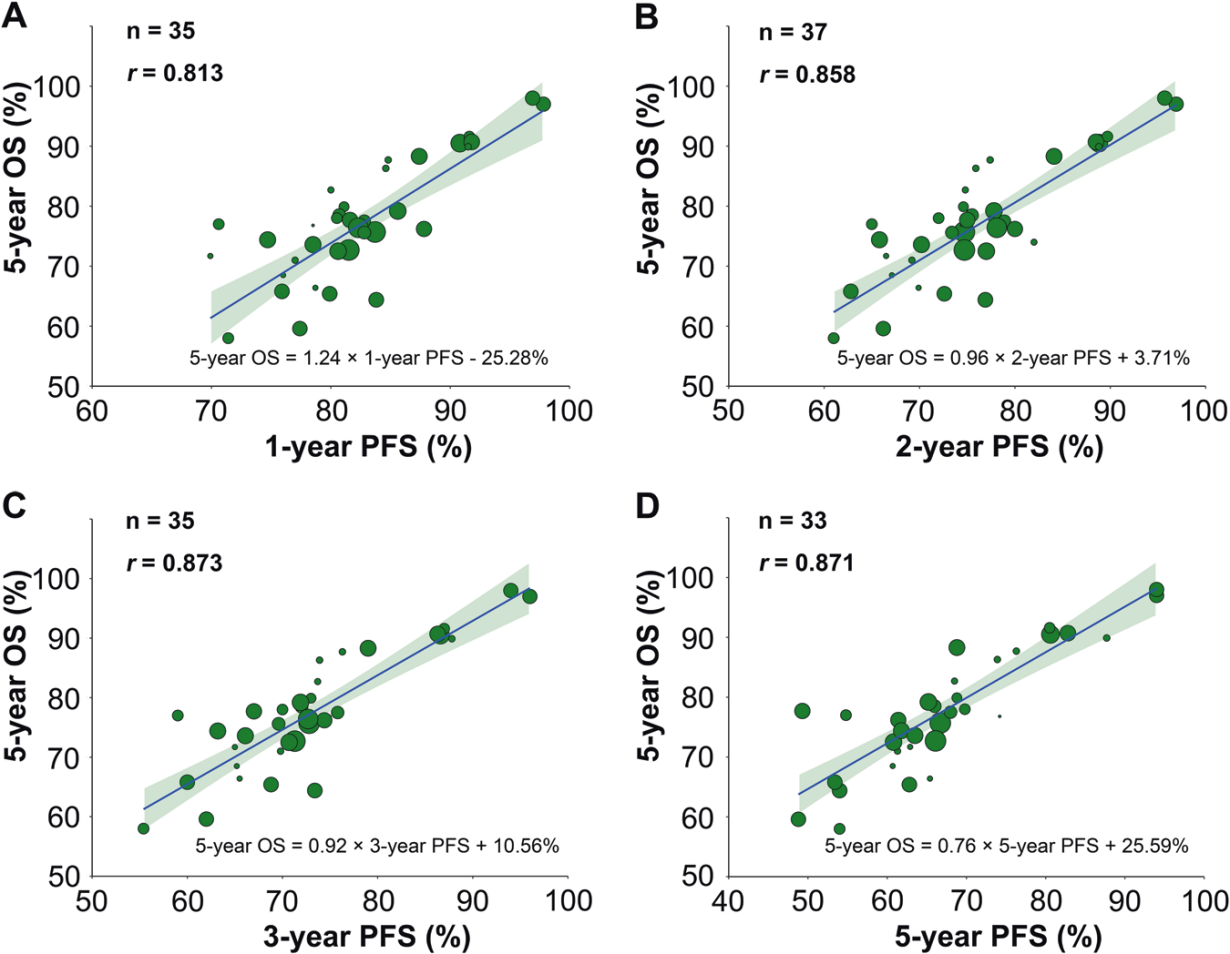
Rituximab Immunochemotherapy Arm-level Correlation Between PFS and OS in RCTs. The rituximab immunochemotherapy arm-level associations between **a** 1-year PFS and 5-year OS, **b** 2-year PFS and 5-year OS, **c** 3-year PFS and 5-year OS, and **d** 5-year PFS and 5-year OS. Circle size is proportional to the number of patients in each treatment arm. The solid blue line indicates the fitted weighted linear regression line; the light green zone represents its 95% CI; *r* indicates the correlation coefficient. PFS progression-free survival; OS overall survival; RCTs randomized controlled trials; CI confidence interval.

Forty-four rituximab immunochemotherapy arms from 26 RCTs reported 5-year OS. Thirty-five (80%) rituximab immunochemotherapy arms reported 1-year and 3-year PFS; 37 (84%) arms reported 2-year PFS and 33 (75%) arms reported 5-year PFS. The 1-year (*r* = 0.813; 95% CI, 0.624–0.913; Fig. 4a), 2-year (*r* = 0.858; 95% CI, 0.705–0.933; Fig. 4b), 3-year (*r* = 0.873; 95% CI, 0.716–0.946; Fig. 4c), or 5-year PFS (*r* = 0.871; 95% CI, 0.711–0.954; Fig. 4d) correlated linearly with 5-year OS. Generally speaking, sensitivity analyses continued to demonstrate robust consistency in terms of correlation *r*. When leaving out 10 trials from R-CHOP (like) with rituximab+intensified/de-escalated chemotherapy subgroup (Supplemental Fig. 1c–f), which account for nearly half of all treatment arms, the findings remain consistent with wider confidence intervals due to the reduced number of arms.

**Fig. 5:**
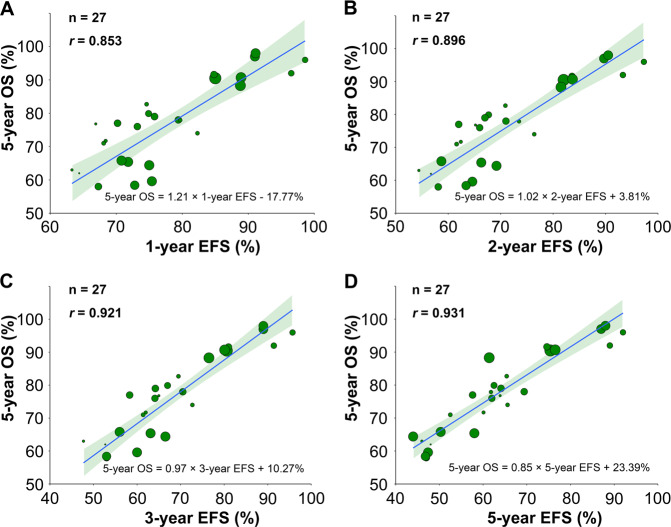
Rituximab Immunochemotherapy Arm-level Correlation Between EFS and OS in RCTs. The rituximab immunochemotherapy arm-level associations between **a** 1-year EFS and 5-year OS, **b** 2-year EFS and 5-year OS, **c** 3-year EFS and 5-year OS, and **d** 5-year EFS and 5-year OS. Circle size is proportional to the number of patients in each treatment arm. The solid blue line indicates the fitted weighted linear regression line; the light green zone represents its 95% CI; *r* indicates the correlation coefficient. EFS event-free survival; OS overall survival; RCTs randomized controlled trials; CI confidence interval.

Twenty-seven rituximab immunochemotherapy arms (61%) reported 1-, 2-, 3- and 5-year EFS. Linear regression analysis revealed correlations between 1-year (*r* = 0.853; 95% CI, 0.729–0.920; Fig. 5a), 2-year (*r* = 0.896; 95% CI, 0.815–0.945; Fig. 5b), 3-year (*r* = 0.921; 95% CI, 0.851–0.966; Fig. 5c), or 5-year EFS (*r* = 0.931; 95% CI, 0.855–0.975; Fig. 5d) and 5-year OS. Sensitivity analysis indicated good consistency (Supplementary Fig. 1g–j). This finding indicates that improvements in 1–3-year PFS or EFS are associated with higher 5-year OS.

### External validation of association of PFS with OS in Phase II and retrospective studies

**Fig. 6:**
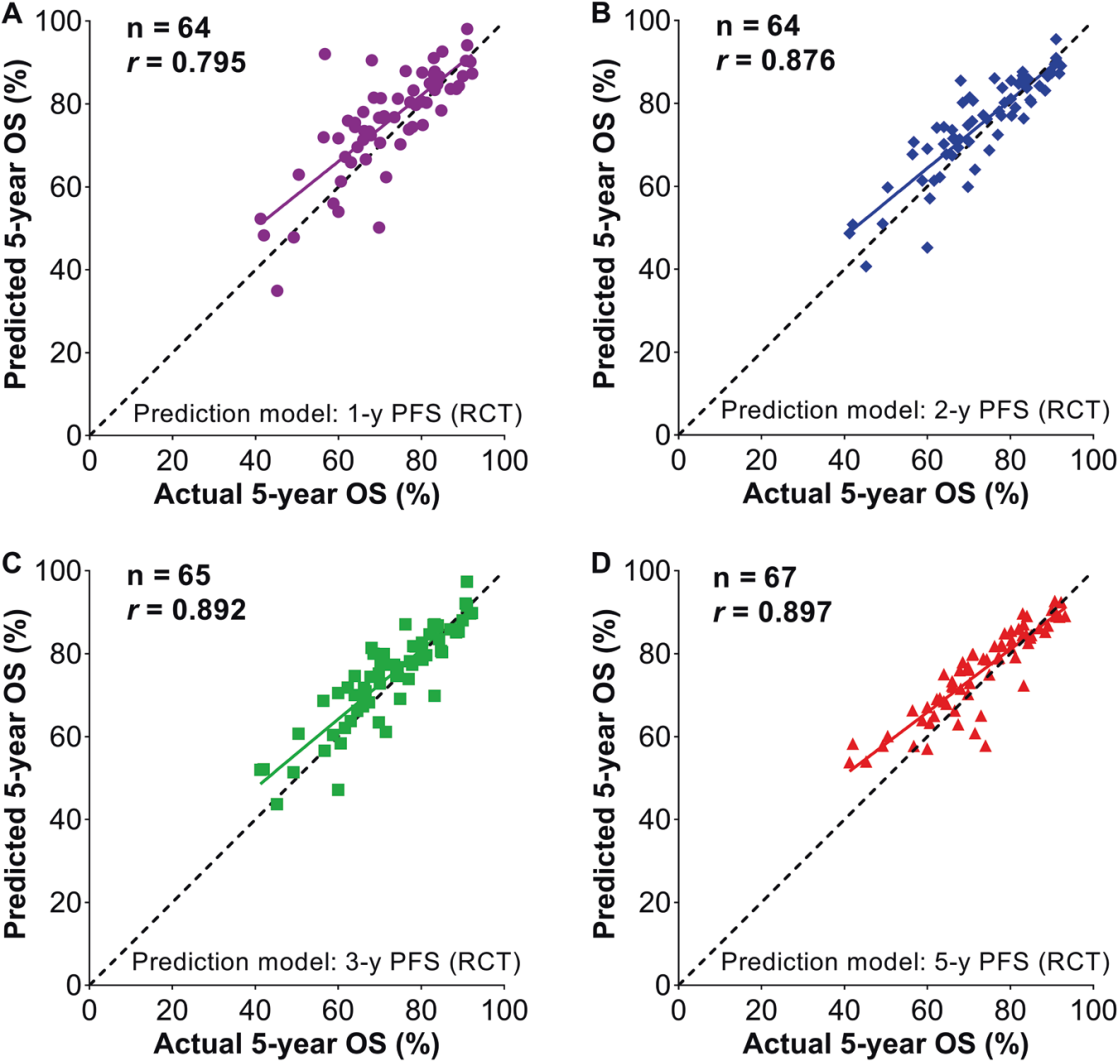
External validation of association of PFS with OS after Rituximab immunochemotherapy. Using PFS linear regression models (as shown in Fig. 4), the predicted 5-year OS, as calculated according to the actual 1-, 2-, 3-, and 5-year PFS from the phase II trials and retrospective data (Table 2), is plotted against the actual 5-year OS. The predicted OS approximates to the actual OS, as indicated by approaching the diagonal line, i.e., the line of identity; *r* indicates the correlation coefficient. PFS progression-free survival; OS overall survival.

Sixty-seven treatment arms from the phase II and retrospective studies were used for external validation. As EFS was not available in the retrospective studies, only PFS prediction models could be evaluated. Using the PFS predictive models from the RCTs (Fig. 4), we calculated the predicted 5-year OS rate for each retrospective study using the actual 1-, 2-, 3-, or 5-year PFS rate (Table 2). The simple regression line between the actual and predicted 5-year OS approached the diagonal line, indicating that the predicted OS was approximated to the actual OS. The predicted 5-year OS rate correlated significantly with the actual 5-year OS rate, with the correlation coefficient *r* ranging from 0.795 to 0.897 (Fig. 6a–d). This finding validates the premise that PFS is predictive of OS.

## Discussion

This is a large-scale, comprehensive study combining data from high-quality phase III RCTs, phase II trials, and retrospective studies to assess the association between the early efficacy endpoints of PFS or EFS with OS in patients with DLBCL primarily treated with immunochemotherapy. Consistent with previous findings [9–12], analyses of the 26 qualified RCTs showed that improved PFS or EFS correlated with OS benefit at trial level. There was a linear correlation between 1–5-year PFS or EFS and 5-year OS rates at treatment arm level. The comprehensive sensitivity analyses indicated an acceptable overall consistency of the developed predictive model across settings. The external validation showed good calibration between the actual and predicted 5-year OS rates based on the 1–5-year PFS rates in the phase II and retrospective studies. These findings provide new evidence supporting the clinical use of PFS and EFS as early efficacy endpoints for evaluating treatment benefit and accelerating approval for superior treatments.

Previous studies, primarily using 13 RCTs conducted before 2015, concluded that the early efficacy endpoints of EFS or PFS are strongly related to OS at both individual and trial level [9–11]. The survival of DLBCL patients who achieved PFS or EFS at 24 months is almost equal to that of the age- and sex-matched general population [9–12]. Therefore, 2-year EFS or PFS are accepted as early efficacy endpoints. Although the use of individual patient data allows better characterization of important covariates that affect survival, it restricts the analysis to a limited number of RCTs, and the analysis is not easily replicated by independent researchers. In most recently published trials and in clinical practice, there are multiple effective agents not only as initial treatment but also in second-line or salvage settings. Any validation of an early efficacy endpoint is relevant only within the context in which the validation occurred. These factors prompted re-examination and external validation of the correlation between PFS or EFS at the given time points with OS. The present literature-based analysis relied on data from RCTs, phase II trials, and retrospective studies to assess the validity of the early efficacy endpoints, and represents a critical step toward understanding the impact of immunochemotherapy on PFS or EFS and OS in DLBCL. With strict inclusion criteria and quality control, we included large-scale, qualified RCTs for trial-level surrogacy analysis, and phase II trials and retrospective studies for external validation. The correlation between PFS or EFS with OS was well established for DLBCL at both the trial and treatment arm level from the RCTs. Furthermore, the correlation between 1–5-year PFS and OS was externally validated by analyzing the phase II and retrospective data. Consistent with previous studies [9–12], these results highlight the significant role of PFS and EFS as early efficacy endpoints in designing prospective trials.

As the association of improved PFS or EFS with prolonged OS in DLBCL in this study is straightforward, the use of PFS and EFS as early efficacy endpoints not only incorporates survival, but also reduces treatment-related events, disease relapse, and progression. Compared with long-term OS, dynamic assessment of PFS or EFS at 1–3 years has a lower likelihood of confounding by subsequent or salvage treatment. Innovative treatment strategies with a large magnitude of effect on PFS or EFS for high-risk patients with DLBCL may have a large effect on OS in RCTs. Importantly, we found that PFS or EFS as early as 1 year correlated with 5-year OS at the treatment arm-level, mainly because the majority of patients were at high risk of early relapse and poor post-progression survival. Consistent with this finding, other studies have demonstrated that ~70% of disease failures occurred within the first year after treatment, but rarely after 5 years [9, 12]. For patients who achieved EFS at 12 and 24 months, the risk of relapse in the next 5 years dropped to 13% and 8%, respectively [9]. If patients experienced progression or relapse within 2 years, the median OS after disease progression was only 7.2 months [11].

The strengths of this study include the quality control design, large sample size, external validation of PFS outcomes, and current standard treatment. First, the data were obtained from high-quality RCTs, phase II, and retrospective studies that enrolled large-scale cohorts (>31,000 patients) with newly diagnosed DLBCL uniformly treated with rituximab-containing immunochemotherapy. We could eliminate selection bias with great confidence due to the limited number of RCTs or treatment option heterogeneity. This comprehensive surrogacy study at trial- and treatment arm-level complements previous evidence and strengthens the clinical use of PFS and EFS as early efficacy endpoints. Second, the positive relationships between the 1–5-year PFS and 5-year OS rates were externally validated using independent data that included patients across different countries with varied eligibility criteria, immunochemotherapy regimens, radiotherapy, and follow-up times. As a variety of immunochemotherapy regimens was investigated in a heterogeneous population, we could examine for variability in treatment outcomes and hence improved the generalizability of our study. Our generation and validation of prediction models for describing the association between the 1–5-year PFS and 5-year OS rates is unique. The RCT validation in an independent cohort improved the reliability of the conclusions.

The study limitations include the lack of individual patient data and standardized definition of endpoints and follow-up assessments. First, this is a literature-based systematic review without individual patient data; therefore, patient-level surrogacy was absent. Second, precise modeling requires standardized definitions of endpoints and standardized follow-up assessments or surveillance strategies in DLBCL trials, which is infeasible to accomplish in our study. For example, while PFS was calculated from the date of randomization in RCTs, it was generally calculated from diagnosis or initial therapy in retrospective studies. In addition, EFS events typically consisted of both PFS events, as well as unplanned treatment, treatment discontinuation and toxic events as they were used to evaluate the safety, toxicity or compliance of a novel therapy. Moreover, EFS events were defined inconsistently across trials and dependent on the trial design and purpose. In clinical practice, the exact date of disease progression is difficult to determine precisely, such that the reported PFS or EFS event date was naturally dependent on the frequency and interval of two consecutive clinical visits and imaging assessments. Such an inherited heterogeneity in the interval and frequency of assessments across cannot be removed nor quantified. Third, the predicted model concluded in this study was based on findings in patients treated with anthracycline-based immunochemotherapy, and its extrapolation to other treatments would be speculative. The impact of post-progression management was beyond the scope of this study, and such information is not routinely collected in clinical trials. When more effective salvage treatment occurs and post-progression survival is significantly prolonged in the future, the predicted model should also be modified and optimized. Fourth, the correlation between EFS and OS was not externally validated in the retrospective populations, because EFS is generally not reported in retrospective studies.

In conclusion, our assessment of a large sample of high-quality data for patients with DLBCL provides high-level evidence that PFS and EFS are valid early efficacy endpoints for OS in the immunochemotherapy era.

## Supplementary information

Supplemental Figure 1

Supplemental Table 1

Supplemental Table 2

Supplemental Table 3

Supplemental Table 4

Supplemental Figure and Table legends
