# Supplementary material for: Association of progression-free or event-free survival with overall survival in diffuse large B-cell lymphoma after immunochemotherapy: a systematic review
Source: Leukemia. 2020 Jul 10;34(10):2576–91. doi: 10.1038/s41375-020-0963-1 (PMC7515849; doi:10.1038/s41375-020-0963-1)
Supplement: Supplementary file 2 — Supplemental Table 1 [file 41375_2020_963_MOESM2_ESM.docx]

**Supplemental Table 1.** The assessment of risk of bias in randomized controlled trials by using the Cochrane Collaboration tool

| Trial | Bias | Risk of bias | Support for judgement |
| --- | --- | --- | --- |
| **R-CHOP (like) vs.** **CHOP (like)** | | | |
| LNH-98.5 (Coiffier, 2010) | Random sequence generation (selection bias) | Unclear risk | Insufficient information about the sequence generation process |
|  | Allocation concealment (selection bias) | Low risk | Central randomization, participants and investigators can’t foresee assignment |
|  | Blinding of participants and personnel (performance bias) | Low risk | Open label. But outcome is not likely to be influenced by lack of blinding. |
|  | Blinding of outcome assessment (detection bias) | Low risk | Open label. But outcome measurement is not likely to be influenced by lack of blinding. |
|  | Incomplete outcome data (attrition bias) | Low risk | The tumor response of 2/202 in R-CHOP and 7/197 in CHOP couldn’t be assessed because of toxic effects, the patient’s decision, or the investigator’s decision before evaluation of the tumor. Comment: similar reasons, unlikely to influence outcome. |
|  | Selective reporting (reporting bias) | Low risk | All of prespecified outcomes were reported. |
|  | Other bias | Low risk | The study appeared to be free of other sources of bias. |
| MInT (Pfreundschuh, 2011) | Random sequence generation (selection bias) | Low risk | Patients were randomized centrally by a data manager at the Intergroup Data Centre using a computer-based randomisation tool with an algorithm that accounted for randomisations that had occurred previously to ensure balanced randomisation at any time. |
|  | Allocation concealment (selection bias) | Low risk | Centres were informed of randomization results by fax. Participants and investigators can’t foresee assignment |
|  | Blinding of participants and personnel (performance bias) | Low risk | Open label. But outcome is not likely to be influenced by lack of blinding. |
|  | Blinding of outcome assessment (detection bias) | Low risk | Open label. But outcome measurement is not likely to be influenced by lack of blinding. |
|  | Incomplete outcome data (attrition bias) | Low risk | 60/410 and 58/413 not evaluated for response, respectively. Comment: similar reasons and numbers, unlikely to influence outcome. |
|  | Selective reporting (reporting bias) | Low risk | All of prespecified outcomes were reported. |
|  | Other bias | Low risk | The study appeared to be free of other sources of bias. |
| ECOG4494/CALGB97933 (Habermann, 2006) | Random sequence generation (selection bias) | Low risk | Use permuted block within stratum to generate random study arm assignment sequence and patients will be randomized accordingly. |
|  | Allocation concealment (selection bias) | low risk | Because of the permuted block and strata information (most likely also study site information) and central registration (see below), the sequence can not be predicted by the patients or study site PIs. We do not sent the allocation sequence to site, rather all patients will randomized centrally using ECOG central registration system. |
|  | Blinding of participants and personnel (performance bias) | Low risk | Open label. But outcome is not likely to be influenced by lack of blinding. |
|  | Blinding of outcome assessment (detection bias) | Low risk | Open label. But outcome measurement is not likely to be influenced by lack of blinding. |
|  | Incomplete outcome data (attrition bias) | Low risk | The concept of the weighted analysis to remove the bias that can result from analyzing only a subset of the patients in two-stage randomized designs, is consistent with previously proposed methods for the missing data problem. |
|  | Selective reporting (reporting bias) | Low risk | All of prespecified outcomes were reported. |
|  | Other bias | Low risk | The study appeared to be free of other sources of bias. |
| RICOVER-60 (Pfreundschuh M, 2008) | Random sequence generation (selection bias) | Low risk | Randomisation was undertaken at a 1:1:1:1 ratio by use of the Pocock minimisation algorithm after stratification for centres. Patients were randomised centrally by a data manager at the study centre by use of a computer program with an algorithm that accounted for randomisations that had occurred previously. |
|  | Allocation concealment (selection bias) | Low risk | Central randomization, participants and investigators can’t foresee assignment |
|  | Blinding of participants and personnel (performance bias) | Low risk | Open label. But outcome is not likely to be influenced by lack of blinding. |
|  | Blinding of outcome assessment (detection bias) | Low risk | Open label. But outcome measurement is not likely to be influenced by lack of blinding. |
|  | Incomplete outcome data (attrition bias) | Low risk | 20 patients were excluded because of missing or retracted informed consent. The trial was stopped on June 14, 2005, and 44 of 1222 patients who were still under treatment at that time were censored on that date. Comment: similar reasons and numbers, unlikely to influence outcome. |
|  | Selective reporting (reporting bias) | Low risk | All of prespecified outcomes were reported. |
|  | Other bias | Low risk | The study appeared to be free of other sources of bias. |
| **R-CHOP (like) vs. R + intensified/de-escalated chemotherapy** | | | |
| LNH03-2B (Récher, 2011) | Random sequence generation (selection bias) | Low risk | Random assignment was done with a computer-assisted randomization allocation sequence with a block size of four. |
|  | Allocation concealment (selection bias) | Low risk | Central randomization, participants and investigators can’t foresee assignment |
|  | Blinding of participants and personnel (performance bias) | Low risk | Open label. But outcome is not likely to be influenced by lack of blinding. |
|  | Blinding of outcome assessment (detection bias) | Low risk | Open label. But outcome measurement is not likely to be influenced by lack of blinding. |
|  | Incomplete outcome data (attrition bias) | Low risk | Only one participant withdrew consent before treatment, which is unlikely to influence outcome. |
|  | Selective reporting (reporting bias) | Low risk | All of prespecified outcomes were reported. |
|  | Other bias | Low risk | The study appeared to be free of other sources of bias. |
| ANZINTER3 (Merli, 2012) | Random sequence generation (selection bias) | Unclear risk | Insufficient information about the sequence generation process available to permit a judgement of ‘low risk’ or ‘high risk’. |
|  | Allocation concealment (selection bias) | Unclear risk | The method of concealment is not described to allow a definite judgement |
|  | Blinding of participants and personnel (performance bias) | Low risk | Open label. But outcome is not likely to be influenced by lack of blinding. |
|  | Blinding of outcome assessment (detection bias) | Low risk | Open label. But outcome measurement is not likely to be influenced by lack of blinding. |
|  | Incomplete outcome data (attrition bias) | Low risk | After randomization, four patients were considered ineligible and were excluded (1 in R-CHOP and 3 in R-miniCEOP). It is unlikely to influence outcome. |
|  | Selective reporting (reporting bias) | Low risk | All of prespecified outcomes were reported. |
| LNH03-6B (Delarue, 2013) | Random sequence generation (selection bias) | Low risk | The trial used computer-assisted permuted-block randomisation (block size of four, allocation ratio 1:1) to assign treatment. |
|  | Allocation concealment (selection bias) | Low risk | Central randomization, and the treatment allocation was sent to the investigator by fax. Participants and investigators can’t foresee assignment. |
|  | Blinding of participants and personnel (performance bias) | Low risk | Open label. But outcome is not likely to be influenced by lack of blinding. |
|  | Blinding of outcome assessment (detection bias) | Low risk | Open label. But outcome measurement is not likely to be influenced by lack of blinding. |
|  | Incomplete outcome data (attrition bias) | Low risk | After randomization, two participants received no treatment and 1 died before treatment (due to concurrent illness). It is unlikely to influence outcome. |
|  | Selective reporting (reporting bias) | Low risk | All of prespecified outcomes were reported. |
|  | Other bias | Low risk | The study appeared to be free of other sources of bias. |
| NCT01793844 (Li, 2019) | Random sequence generation (selection bias) | Low risk | Patients were centrally, randomly assigned to treatment with R-CHOP-14 or R-CHOP-21 in a 1:1 ratio by an independent statistician using a computer-generated randomization schedule. |
|  | Allocation concealment (selection bias) | Low risk | Central randomization. The randomization code was provided in sealed envelopes. Participants and investigators can’t foresee assignment. |
|  | Blinding of participants and personnel (performance bias) | Low risk | Open label. But outcome is not likely to be influenced by lack of blinding. |
|  | Blinding of outcome assessment (detection bias) | Low risk | Open label. But outcome measurement is not likely to be influenced by lack of blinding. |
|  | Incomplete outcome data (attrition bias) | Low risk | 6/349 and 9/353 participants withdrew consent in R-CHOP-14 and R-CHOP-21 group, respectively. |
|  | Selective reporting (reporting bias) | Low risk | All of prespecified outcomes were reported. |
|  | Other bias | Low risk | The study appeared to be free of other sources of bias. |
| UK NCRI (Cunningham, 2013) | Random sequence generation (selection bias) | Low risk | Randomisation was done centrally by the Cancer Research UK and University College London Cancer Trials Centre, London, UK, using a minimisation procedure, stratified for international prognostic index and centre. |
|  | Allocation concealment (selection bias) | Low risk | Central randomization, participants and investigators can’t foresee assignment. |
|  | Blinding of participants and personnel (performance bias) | Low risk | Open label. But outcome is not likely to be influenced by lack of blinding. |
|  | Blinding of outcome assessment (detection bias) | Low risk | Open label. But outcome measurement is not likely to be influenced by lack of blinding. |
|  | Incomplete outcome data (attrition bias) | Low risk | After randomization, 5/540 and 4/540 participants were excluded for similar reasons. 35/535 and 34/536 participants were not assessed for end-of-treatment response. |
|  | Selective reporting (reporting bias) | Low risk | All of prespecified outcomes were reported. |
|  | Other bias | Low risk | The study appeared to be free of other sources of bias. |
| DLCL04 (Chiappella, 2017) | Random sequence generation (selection bias) | Low risk | The randomisation sequence was generated by the statistician by use of a computer program and implemented by means of a Web-based procedure, which was concealed to researchers. |
|  | Allocation concealment (selection bias) | Low risk | Central randomization, participants and investigators can’t foresee assignment. |
|  | Blinding of participants and personnel (performance bias) | Low risk | Open label. But outcome is not likely to be influenced by lack of blinding. |
|  | Blinding of outcome assessment (detection bias) | Low risk | Open label. But outcome measurement is not likely to be influenced by lack of blinding. |
|  | Incomplete outcome data (attrition bias) | Low risk | No participants were excluded after randomization. |
|  | Selective reporting (reporting bias) | Low risk | All of prespecified outcomes were reported. |
|  | Other bias | Low risk | The study appeared to be free of other sources of bias. |
| Alliance/CALGB 50303 (Bartlett, 2019) | Random sequence generation (selection bias) | Low risk | “It was a standard statistical randomization. Investigators had no influence on randomization.” by the email from the corresponding author. |
|  | Allocation concealment (selection bias) | Low risk | “It was a standard statistical randomization. Investigators had no influence on randomization.” by the email from the corresponding author. |
|  | Blinding of participants and personnel (performance bias) | Low risk | Open label. But outcome is not likely to be influenced by lack of blinding. |
|  | Blinding of outcome assessment (detection bias) | Low risk | Open label. But outcome measurement is not likely to be influenced by lack of blinding. |
|  | Incomplete outcome data (attrition bias) | Low risk | After randomization, 12/262 and 21/262 participants were excluded for similar reasons. |
|  | Selective reporting (reporting bias) | Low risk | All of prespecified outcomes were reported. |
|  | Other bias | Low risk | The study appeared to be free of other sources of bias. |
| FLYER (Poeschel, 2019) | Random sequence generation (selection bias) | Low risk | Randomization was done in a 1:1 ratio using the Pocock minimization algorithm after stratification for centers, stage (Ann Arbor stage I vs II) and extralymphatic sites (no vs yes). To ensure balanced group assignment at any time, patients were randomly assigned centrally by a data manager at the study center (Homburg, Germany) by use of a computer program with an algorithm using a biased coin approach that accounted for previous randomizations. |
|  | Allocation concealment (selection bias) | Low risk | Central randomization, participants and investigators can’t foresee assignment. |
|  | Blinding of participants and personnel (performance bias) | Low risk | Open label. But outcome is not likely to be influenced by lack of blinding. |
|  | Blinding of outcome assessment (detection bias) | Low risk | Open label. But outcome measurement is not likely to be influenced by lack of blinding. |
|  | Incomplete outcome data (attrition bias) | Low risk | After randomization, 4/297 participants were excluded for withdrawing consent in R-CHOP + R*2 group. 53/293 and 53/295 participants were excluded form ITT group, because of no reference pathology available or meeting the exclusion criteria. |
|  | Selective reporting (reporting bias) | Low risk | All of prespecified outcomes were reported. |
|  | Other bias | Low risk | The study appeared to be free of other sources of bias. |
| PETAL (Dührsen, 2018) | Random sequence generation (selection bias) | Low risk | Central randomization within each group using the Pocock and Simon minimization method. |
|  | Allocation concealment (selection bias) | Low risk | Central randomization, participants and investigators can’t foresee assignment. |
|  | Blinding of participants and personnel (performance bias) | Low risk | Open label. But outcome is not likely to be influenced by lack of blinding. |
|  | Blinding of outcome assessment (detection bias) | Low risk | Open label. But outcome measurement is not likely to be influenced by lack of blinding. |
|  | Incomplete outcome data (attrition bias) | Low risk | No participants were excluded after randomization. |
|  | Selective reporting (reporting bias) | Low risk | All of prespecified outcomes were reported. |
|  | Other bias | Low risk | The study appeared to be free of other sources of bias. |
| NHL-001 (Xu, 2019) | Random sequence generation (selection bias) | Low risk | We used computer-assisted permuted-block randomisation—with a block size of six  and allocation ratio of 1:1:1 (R-CHOP50, R-CEOP70, or R-CEOP90) for patients 60 years and younger, and a block size of four and allocation ratio of 1:1 (R-CHOP50 or R-CEOP70) for patients older than 60 years—to assign treatment. |
|  | Allocation concealment (selection bias) | Low risk | Central randomization, participants and investigators can’t foresee assignment. |
|  | Blinding of participants and personnel (performance bias) | Low risk | Open label. But outcome is not likely to be influenced by lack of blinding. |
|  | Blinding of outcome assessment (detection bias) | Low risk | Open label. But outcome measurement is not likely to be influenced by lack of blinding. |
|  | Incomplete outcome data (attrition bias) | Low risk | A total of 4 participants withdrew consent and 1 was misdiagnosed after randomization. |
|  | Selective reporting (reporting bias) | Low risk | All of prespecified outcomes were reported. |
|  | Other bias | Low risk | The study appeared to be free of other sources of bias. |
| **R-CHOP (like) chemotherapy followed by maintenance/consolidation therapy** | | | |
| AGMT-NHL13  (Jaeger, 2015) | Random sequence generation (selection bias) | Unclear risk | Insufficient information about the sequence generation process available to permit a judgement of ‘low risk’ or ‘high risk’. |
|  | Allocation concealment (selection bias) | Unclear risk | The method of concealment is not described to allow a definite judgement |
|  | Blinding of participants and personnel (performance bias) | Low risk | Open label. But outcome is not likely to be influenced by lack of blinding. |
|  | Blinding of outcome assessment (detection bias) | Low risk | Open label. But outcome measurement is not likely to be influenced by lack of blinding. |
|  | Incomplete outcome data (attrition bias) | Low risk | Similar number of patients and similar reasons between maintenance and observation group. |
|  | Selective reporting (reporting bias) | Low risk | All of prespecified outcomes were reported. |
|  | Other bias | Low risk | The study appeared to be free of other sources of bias. |
| PRELUDE (Crump, 2016) | Random sequence generation (selection bias) | Unclear risk | Insufficient information about the sequence generation process available to permit a judgement of ‘low risk’ or ‘high risk’. |
|  | Allocation concealment (selection bias) | Unclear risk | The method of concealment is not described to allow a definite judgement |
|  | Blinding of participants and personnel (performance bias) | Low risk | Double-blind design. |
|  | Blinding of outcome assessment (detection bias) | Low risk | It is unknown whether outcome assessor was blind. But outcome measurement is not likely to be influenced by lack of blinding. |
|  | Incomplete outcome data (attrition bias) | Low risk | 11 participants did not receive enzastaurin and 2 lost to follow up in enzastaurin group. 5 participants did not receive enzastaurin in enzastaurin group. |
|  | Selective reporting (reporting bias) | Low risk | All of prespecified outcomes were reported. |
|  | Other bias | Low risk | The study appeared to be free of other sources of bias. |
| REMARC (Thieblemont, 2017) | Random sequence generation (selection bias) | Unclear risk | Insufficient information about the sequence generation process available to permit a judgement of ‘low risk’ or ‘high risk’. |
|  | Allocation concealment (selection bias) | Unclear risk | The method of concealment is not described to allow a definite judgement |
|  | Blinding of participants and personnel (performance bias) | Low risk | Double-blind design. |
|  | Blinding of outcome assessment (detection bias) | Low risk | It is unknown whether outcome assessor was blind. But outcome measurement is not likely to be influenced by lack of blinding. |
|  | Incomplete outcome data (attrition bias) | Low risk | After randomization, 11/323 and 5/327 participants withdrew consent after randomization. |
|  | Selective reporting (reporting bias) | Low risk | All of prespecified outcomes were reported. |
|  | Other bias | Low risk | The study appeared to be free of other sources of bias. |
| PILLAR-2 (Witzig, 2018) | Random sequence generation (selection bias) | Low risk | “The random sequence was at Novartis and I am sure it was computer generated. There was no way that any of us researchers or patients could predict. The allocation was sent to the study site electronically.” by the email from the corresponding author. |
|  | Allocation concealment (selection bias) | Low risk | “The random sequence was at Novartis and I am sure it was computer generated. There was no way that any of us researchers or patients could predict. The allocation was sent to the study site electronically.” by the email from the corresponding author. |
|  | Blinding of participants and personnel (performance bias) | Low risk | Open label. But outcome is not likely to be influenced by lack of blinding. |
|  | Blinding of outcome assessment (detection bias) | Low risk | Open label. But outcome measurement is not likely to be influenced by lack of blinding. |
|  | Incomplete outcome data (attrition bias) | Low risk | Eighteen participants withdrew consent and 1 participant was lost to follow-up in everolimus group. Seven participants withdrew consent and 2 participants were lost to follow-up in placebo group. |
|  | Selective reporting (reporting bias) | Low risk | All of prespecified outcomes were reported. |
|  | Other bias | Low risk | The study appeared to be free of other sources of bias. |
| DSHNHL2002-1 (Schmitz, 2012) | Random sequence generation (selection bias) | Low risk | Randomization was done in a 1:1 ratio with the Pocock minimization algorithm at the Institute for Medical Informatics, Statistics, and Epidemiology in Leipzig, Germany. |
|  | Allocation concealment (selection bias) | Low risk | Central randomization, participants and investigators can’t foresee assignment. |
|  | Blinding of participants and personnel (performance bias) | Low risk | Open label. But outcome is not likely to be influenced by lack of blinding. |
|  | Blinding of outcome assessment (detection bias) | Low risk | Open label. But outcome measurement is not likely to be influenced by lack of blinding. |
|  | Incomplete outcome data (attrition bias) | Low risk | 6/136 and 7/139 were excluded for withdrawing consent or missing data. Comment: similar reasons and numbers, unlikely to influence outcome. |
|  | Selective reporting (reporting bias) | Low risk | All of prespecified outcomes were reported. |
|  | Other bias | Low risk | The study appeared to be free of other sources of bias. |
| NCT00355199 (Cortelazzo, 2016) | Random sequence generation (selection bias) | Low risk | Randomization was centralized at Mario Negri Sud Research Foundation through a Web-based system. |
|  | Allocation concealment (selection bias) | Low risk | Central randomization, participants and investigators can’t foresee assignment. |
|  | Blinding of participants and personnel (performance bias) | Low risk | Open label. But outcome is not likely to be influenced by lack of blinding. |
|  | Blinding of outcome assessment (detection bias) | Low risk | Open label. But outcome measurement is not likely to be influenced by lack of blinding. |
|  | Incomplete outcome data (attrition bias) | Low risk | After randomization, 4/126 and 7/120 was excluded for similar reason. One patient was lost to follow-up in R-HDS group. |
|  | Selective reporting (reporting bias) | Low risk | All of prespecified outcomes were reported. |
|  | Other bias | Low risk | The study appeared to be free of other sources of bias. |
| LYSA/GOELAMS (Lamy, 2018) | Random sequence generation (selection bias) | Unclear risk | Insufficient information about the sequence generation process available to permit a judgement of ‘low risk’ or ‘high risk’. |
|  | Allocation concealment (selection bias) | Low risk | Central randomization, participants and investigators can’t foresee assignment. |
|  | Blinding of participants and personnel (performance bias) | Low risk | Open label. But outcome is not likely to be influenced by lack of blinding. |
|  | Blinding of outcome assessment (detection bias) | Low risk | Open label. But outcome measurement is not likely to be influenced by lack of blinding. |
|  | Incomplete outcome data (attrition bias) | Low risk | 6/165 and 9/169 were excluded for similar reasons. |
|  | Selective reporting (reporting bias) | Low risk | All of prespecified outcomes were reported. |
|  | Other bias | Low risk | The study appeared to be free of other sources of bias. |
| **R-CHOP + novel targeted drug** | | | |
| MAIN (Seymour, 2014) | Random sequence generation (selection bias) | Low risk | “Blinded, central randomization notified by email-NOT predictable by researcher or patient” by the email from the corresponding author. |
|  | Allocation concealment (selection bias) | Low risk | “Blinded, central randomization notified by email-NOT predictable by researcher or patient” by the email from the corresponding author. |
|  | Blinding of participants and personnel (performance bias) | Low risk | Double-blind (Participant, Investigator) |
|  | Blinding of outcome assessment (detection bias) | Low risk | It is unknown whether outcome assessor was also blinded. But outcome measurement is not likely to be influenced by lack of blinding. |
|  | Incomplete outcome data (attrition bias) | Low risk | 5 participants withdrew and 24 were lost to follow-up in R-CHOP group (n = 397). 1 participant withdrew and 33 were lost to follow-up in RA-CHOP group (n = 390). |
|  | Selective reporting (reporting bias) | Low risk | All of prespecified outcomes were reported. |
|  | Other bias | Low risk | The study appeared to be free of other sources of bias. |
| REMoDL-B (Davies, 2019) | Random sequence generation (selection bias) | Low risk | Participants were centrally randomly assigned (1:1) with block randomisation of varying block size by TENALEA, a web-based system, to receive either R-CHOP (control) or RB-CHOP (experimental). |
|  | Allocation concealment (selection bias) | Low risk | Central randomization, participants and investigators can’t foresee assignment. |
|  | Blinding of participants and personnel (performance bias) | Low risk | Open label. But outcome is not likely to be influenced by lack of blinding. |
|  | Blinding of outcome assessment (detection bias) | Low risk | Open label. But outcome measurement is not likely to be influenced by lack of blinding. |
|  | Incomplete outcome data (attrition bias) | Low risk | 4/158, 1/244, 5/123, 6/240, 12/235, 1/98 and 6/101 participants were lost to follow-up in each group, respectively. |
|  | Selective reporting (reporting bias) | Low risk | All of prespecified outcomes were reported. |
|  | Other bias | Low risk | The study appeared to be free of other sources of bias. |
| PHOENIX (Younes, 2019) | Random sequence generation (selection bias) | Low risk | Random assignment was based on a computer-generated preplanned schedule, balanced by permuted blocks and stratified by revised International Prognostic Index (1 to 2 v 3 to 5), region (United States or Western Europe v rest of world), and prespecified R-CHOP cycle numbers. |
|  | Allocation concealment (selection bias) | Low risk | Central randomization, participants and investigators can’t foresee assignment. |
|  | Blinding of participants and personnel (performance bias) | Low risk | Double-blind |
|  | Blinding of outcome assessment (detection bias) | Low risk | It is unknown whether outcome assessor was also blinded. But outcome measurement is not likely to be influenced by lack of blinding. |
|  | Incomplete outcome data (attrition bias) | Low risk | Two participants withdrew consent and 5 participants were lost to follow-up in ibrutinib+R-CHOP groups. One participant withdrew consent and 9 participants were lost to follow-up in placebo+R-CHOP groups. |
|  | Selective reporting (reporting bias) | Low risk | All of prespecified outcomes were reported. |
|  | Other bias | Low risk | The study appeared to be free of other sources of bias. |
| **Anti-CD20 monoclonal antibody study** | | | |
| MabEase (Lugtenburg, 2017) | Random sequence generation (selection bias) | Low risk | Patients were randomized 2:1 via a centralized interactive voice/web response system to receive rituximab SC or IV, and were stratified according to age, IPI risk category, and chemotherapy regimen. |
|  | Allocation concealment (selection bias) | Low risk | Central randomization, participants and investigators can’t foresee assignment. |
|  | Blinding of participants and personnel (performance bias) | Low risk | Open label. But outcome is not likely to be influenced by lack of blinding. |
|  | Blinding of outcome assessment (detection bias) | Low risk | Open label. But outcome measurement is not likely to be influenced by lack of blinding. |
|  | Incomplete outcome data (attrition bias) | Low risk | Twenty-one, 21, 20 and 9 participants in rituximab SC group (n =381) withdrew consent, were lost to follow-up, missing and lack of compliance. Fifteen, 17, 5 and 3 participants in rituximab IV group (n =195) withdrew consent, were lost to follow-up, missing and lack of compliance. |
|  | Selective reporting (reporting bias) | Low risk | All of prespecified outcomes were reported. |
|  | Other bias | Low risk | The study appeared to be free of other sources of bias. |
| GOYA (Vitolo, 2017) | Random sequence generation (selection bias) | Low risk | “Randomization will be performed through the IVRS using stratified permuted block randomization. IVRS is a website dedicated to the study and CSRs.” by the email from the corresponding author. |
|  | Allocation concealment (selection bias) | Low risk | “Randomization could not predict at all by researchers or patients. The allocation results were available through IVRS and a confirmation email was also sent automatically by the system. The results were strictly blinded to the investigators, patients and to myself and the steering committee” by the email from the corresponding author. |
|  | Blinding of participants and personnel (performance bias) | Low risk | Open label. But outcome is not likely to be influenced by lack of blinding. |
|  | Blinding of outcome assessment (detection bias) | Low risk | Open label. But outcome measurement is not likely to be influenced by lack of blinding. |
|  | Incomplete outcome data (attrition bias) | Low risk | In R-CHOP group (n = 712), 8 participants received no treatment. 1 with noncompliance, 8 withdrew consent, and 9 was lost to follow-up. In G-CHOP group (n = 706), 3 participants received no treatment. 1 with noncompliance, 3 withdrew consent, and 10 was lost to follow-up. |
|  | Selective reporting (reporting bias) | Low risk | All of prespecified outcomes were reported. |
|  | Other bias | Low risk | The study appeared to be free of other sources of bias. |
| **Trials with high risk of bias** | | | |
| LNH03-1B (Ketterer, 2012) | Random sequence generation (selection bias) | Unclear risk | Insufficient information about the sequence generation process available to permit a judgement of ‘low risk’ or ‘high risk’. |
|  | Allocation concealment (selection bias) | Unclear risk | The method of concealment is not described to allow a definite judgement |
|  | Blinding of participants and personnel (performance bias) | Low risk | Open label. But outcome is not likely to be influenced by lack of blinding. |
|  | Blinding of outcome assessment (detection bias) | Low risk | Open label. But outcome measurement is not likely to be influenced by lack of blinding. |
|  | Incomplete outcome data (attrition bias) | Low risk | 2/112 and 1/110 was not available for response evaluation. |
|  | Selective reporting (reporting bias) | Low risk | All of prespecified outcomes were reported. |
|  | Other bias | High risk | It should enroll more than 400 participants according to the statistical requirement. But the study only included 223 participants due to a slow rate of inclusion. We tend to believe the insufficient sample size would have a high risk to introduce bias and lead to unreliable results. |
| **Phase II RCT in external validation** | | | |
| LNH2007-3B (Casasnovas, 2017) | Random sequence generation (selection bias) | Unclear risk | Insufficient information about the sequence generation process available to permit a judgement of ‘low risk’ or ‘high risk’. |
|  | Allocation concealment (selection bias) | Unclear risk | The method of concealment is not described to allow a definite judgement |
|  | Blinding of participants and personnel (performance bias) | Low risk | Open label. But outcome is not likely to be influenced by lack of blinding. |
|  | Blinding of outcome assessment (detection bias) | Low risk | Open label. But outcome measurement is not likely to be influenced by lack of blinding. |
|  | Incomplete outcome data (attrition bias) | Low risk | Five participants were excluded for investigator’s decision or unavailable PET in R-ACVBP group (n = 114). Six participants were excluded for unavailable PET in R-CHOP group (n = 108). |
|  | Selective reporting (reporting bias) | Low risk | All of prespecified outcomes were reported. |
|  | Other bias | Low risk | The study appeared to be free of other sources of bias. |

Abbreviation: CHOP, cyclophosphamide, doxorubicin, vincristine, and prednisone; ECOG, Eastern Cooperative Oncology Group; G-CHOP, obinutuzumab, cyclophosphamide, doxorubicin, vincristine and prednisone; IPI, International Prognostic Index; ITT, intent-to-treat; IV, intravenous; PET, positron emission tomography; R, rituximab; R-ACVBP, rituximab, doxorubicin, cyclophosphamide, vindesine, bleomycin, and prednisone; R-CEOP70, rituximab, cyclophosphamide, epirubicin (70 mg/m2), vincristine, and prednisone; R-CEOP90, rituximab, cyclophosphamide, epirubicin (90 mg/m2), vincristine, and prednisone; R-CHOP, rituximab, cyclophosphamide, doxorubicin, vincristine and prednisone; R-CHOP50, rituximab, cyclophosphamide, doxorubicin (50 mg/m2), vincristine, and prednisone; R-CHOP-14, R-CHOP every 14 days; R-CHOP-21, R-CHOP every 21 days; R-miniCEOP, rituximab, cyclophosphamide, epirubicin, vinblastine, and prednisone; RA-CHOP, R-CHOP with bevacizumab; RB-CHOP, R-CHOP with bortezomib; SC, subcutaneous
