# Supplementary material for: Association of progression-free or event-free survival with overall survival in diffuse large B-cell lymphoma after immunochemotherapy: a systematic review
Source: Leukemia. 2020 Jul 10;34(10):2576–91. doi: 10.1038/s41375-020-0963-1 (PMC7515849; doi:10.1038/s41375-020-0963-1)
Supplement: Supplementary file 3 — Supplemental Table 2 [file 41375_2020_963_MOESM3_ESM.docx]

**Supplemental Table 2.** The assessment of risk of bias in non-randomized trials and retrospective studies by using the Newcastle – Ottawa quality assessment scale for cohort study

| Study | Selection | | | | Comparability | | Outcome (Median follow-up ≥ 2.5 year to get 1 star; follow-up rate > 75% to get 1 star) | | | Total score | Inclusion |
| --- | --- | --- | --- | --- | --- | --- | --- | --- | --- | --- | --- |
|  | Part 1 | Part 2 | Part 3 | Part 4 | Part 1 | Part 2 | Part 1 | Part 2 | Part 3 |  |  |
| **Non-randomized phase II trial (n = 3)** | | | | | | | | | | | |
| DENSE-R-CHOP-14 (Murawski, 2014) | 1 | 1 | 1 | 1 | 1 | 1 | 1 | 1 | 1 | 9 | Yes |
| LNH2003-3 (Fitoussi, 2011) | 1 | 1 | 1 | 1 | 1 | 1 | 1 | 1 | 1 | 9 | Yes |
| Niitsu, 2010 | 1 | 1 | 1 | 1 | 1 | 1 | 1 | 1 | 1 | 9 | Yes |
| **Retrospective study (n = 57)** | | | | | | | | | | | |
| Go, 2019 | 1 | 1 | 1 | 0 | 1 | 1 | 1 | 1 | 0 | 7 | Yes |
| Kaneda, 2019 | 0 | 0 | 0 | 0 | 1 | 1 | 0 | 0 | 0 | 2 | No |
| Lee, 2019 | 1 | 1 | 1 | 0 | 1 | 1 | 1 | 0 | 1 | 7 | Yes |
| Liu, 2019 | 1 | 1 | 0 | 0 | 0 | 0 | 0 | 1 | 0 | 3 | No |
| Morrison, 2019 | 1 | 1 | 1 | 0 | 1 | 1 | 1 | 0 | 1 | 7 | Yes |
| Yim, 2019 | 1 | 1 | 1 | 0 | 1 | 1 | 1 | 1 | 1 | 8 | Yes |
| Chen, 2018 | 1 | 1 | 1 | 0 | 1 | 1 | 0 | 1 | 0 | 6 | Yes |
| Horvat, 2018 | 1 | 1 | 1 | 0 | 0 | 0 | 1 | 1 | 0 | 5 | No |
| Hosoda, 2018 | 1 | 1 | 1 | 0 | 1 | 1 | 1 | 1 | 0 | 7 | Yes |
| Kim, 2018 | 1 | 1 | 1 | 0 | 1 | 1 | 1 | 1 | 0 | 7 | Yes |
| Li, 2018 | 1 | 1 | 1 | 0 | 1 | 1 | 1 | 0 | 1 | 7 | Yes |
| Li, 2018 | 1 | 1 | 1 | 0 | 1 | 1 | 1 | 0 | 1 | 7 | Yes |
| Matsumoto, 2018 | 1 | 1 | 1 | 0 | 1 | 1 | 1 | 1 | 0 | 7 | Yes |
| Novo, 2018 | 0 | 0 | 1 | 0 | 1 | 1 | 0 | 1 | 1 | 5 | No |
| Sun, 2018 | 1 | 1 | 1 | 0 | 1 | 1 | 1 | 1 | 0 | 7 | Yes |
| Yhim, 2018 | 0 | 0 | 1 | 0 | 1 | 1 | 0 | 1 | 0 | 4 | No |
| Ashby, 2017 | 1 | 0 | 1 | 0 | 0 | 0 | 1 | 1 | 0 | 4 | No |
| Castellino, 2017 | 0 | 0 | 0 | 0 | 0 | 0 | 0 | 1 | 0 | 1 | No |
| Go, 2017 | 1 | 1 | 1 | 0 | 1 | 1 | 1 | 1 | 0 | 7 | Yes |
| Kanemasa, 2017 | 1 | 1 | 1 | 0 | 1 | 1 | 1 | 1 | 0 | 7 | Yes |
| Li, 2017 | 1 | 1 | 1 | 0 | 1 | 1 | 1 | 1 | 0 | 7 | Yes |
| Liu, 2017 | 1 | 1 | 1 | 0 | 1 | 1 | 1 | 1 | 0 | 7 | Yes |
| Park, 2017 | 1 | 1 | 1 | 0 | 1 | 1 | 1 | 1 | 0 | 7 | Yes |
| Song, 2017 | 1 | 1 | 1 | 0 | 1 | 1 | 1 | 1 | 0 | 7 | Yes |
| Teranaka, 2017 | 1 | 1 | 1 | 0 | 1 | 1 | 0 | 0 | 0 | 5 | No |
| Tsuyama, 2017 | 1 | 1 | 1 | 0 | 1 | 1 | 1 | 0 | 0 | 6 | Yes |
| Yang, 2017 | 1 | 1 | 1 | 0 | 0 | 0 | 1 | 0 | 0 | 4 | No |
| Alinari, 2016 | 1 | 1 | 1 | 0 | 1 | 1 | 1 | 1 | 0 | 7 | Yes |
| Prochazka, 2016 | 1 | 1 | 1 | 0 | 1 | 1 | 1 | 0 | 0 | 6 | Yes |
| Seo, 2016 | 1 | 1 | 1 | 0 | 1 | 1 | 1 | 1 | 0 | 7 | Yes |
| Dabaja, 2015 | 1 | 1 | 1 | 0 | 1 | 1 | 1 | 1 | 0 | 7 | Yes |
| El-Galaly, 2015 | 1 | 1 | 1 | 0 | 1 | 1 | 1 | 0 | 0 | 6 | Yes |
| Gong, 2015 | 1 | 1 | 1 | 0 | 1 | 1 | 1 | 1 | 0 | 7 | Yes |
| Jurczak, 2015 | 1 | 1 | 1 | 0 | 1 | 0 | 1 | 0 | 0 | 5 | No |
| Kumar, 2015 | 1 | 1 | 1 | 0 | 1 | 1 | 1 | 1 | 0 | 7 | Yes |
| Melchardt, 2015 | 1 | 1 | 1 | 0 | 1 | 1 | 0 | 1 | 0 | 6 | Yes |
| Nakajima, 2015 | 1 | 1 | 1 | 0 | 1 | 1 | 1 | 1 | 0 | 7 | Yes |
| Dabaja, 2014 | 1 | 1 | 1 | 0 | 1 | 1 | 1 | 1 | 0 | 7 | Yes |
| Mian, 2014 | 1 | 1 | 1 | 0 | 1 | 1 | 1 | 0 | 0 | 6 | Yes |
| Castillo, 2013 | 1 | 1 | 1 | 0 | 1 | 1 | 1 | 1 | 1 | 8 | Yes |
| Hasimoto, 2013 | 1 | 1 | 1 | 0 | 1 | 1 | 1 | 0 | 0 | 6 | Yes |
| Kojima, 2013 | 1 | 1 | 1 | 0 | 1 | 1 | 1 | 1 | 0 | 7 | Yes |
| Lu, 2013 | 1 | 1 | 1 | 0 | 1 | 0 | 1 | 1 | 0 | 6 | Yes |
| Ozbalak, 2013 | 1 | 1 | 1 | 0 | 1 | 1 | 1 | 1 | 1 | 8 | Yes |
| Shi, 2013 | 1 | 1 | 1 | 0 | 1 | 1 | 1 | 1 | 0 | 7 | Yes |
| Tomita, 2013 | 1 | 1 | 1 | 0 | 1 | 1 | 1 | 1 | 0 | 7 | Yes |
| Castillo, 2012 | 1 | 1 | 1 | 0 | 1 | 1 | 1 | 0 | 0 | 6 | Yes |
| Huang, 2012 | 1 | 1 | 1 | 0 | 1 | 0 | 1 | 1 | 0 | 6 | Yes |
| Li, 2012 | 1 | 1 | 1 | 0 | 1 | 1 | 1 | 0 | 0 | 6 | Yes |
| Li, 2012 | 1 | 1 | 1 | 0 | 1 | 1 | 1 | 1 | 0 | 7 | Yes |
| Lin, 2012 | 1 | 1 | 1 | 0 | 1 | 1 | 1 | 0 | 0 | 6 | Yes |
| Tomita, 2012 | 1 | 1 | 1 | 0 | 1 | 1 | 1 | 1 | 0 | 7 | Yes |
| Sehn, 2011 | 1 | 1 | 1 | 0 | 1 | 1 | 1 | 1 | 0 | 7 | Yes |
| Bari, 2010 | 1 | 1 | 1 | 0 | 1 | 1 | 1 | 1 | 0 | 7 | Yes |
| Ennishi, 2010 | 1 | 1 | 1 | 0 | 1 | 1 | 1 | 1 | 0 | 7 | Yes |
| Phan, 2010 | 1 | 1 | 1 | 0 | 1 | 1 | 1 | 1 | 0 | 7 | Yes |
| Scandurra, 2010 | 1 | 1 | 1 | 0 | 1 | 1 | 1 | 0 | 0 | 6 | Yes |
