# Supplementary material for: Association of progression-free or event-free survival with overall survival in diffuse large B-cell lymphoma after immunochemotherapy: a systematic review
Source: Leukemia. 2020 Jul 10;34(10):2576–91. doi: 10.1038/s41375-020-0963-1 (PMC7515849; doi:10.1038/s41375-020-0963-1)
Supplement: Supplementary file 4 — Supplemental Table 3 [file 41375_2020_963_MOESM4_ESM.docx]

**Supplemental Table 3.** The definition of progression-free survival and event-free survival and follow-up strategy in phase III randomized controlled trials.

| Trial | PFS | | EFS | | Follow-up strategy | |
| --- | --- | --- | --- | --- | --- | --- |
|  | Starting point | Event | Starting point | Event | Intensity | Method |
| LNH-98.5 (Coiffier, 2010) | Randomization | Identical to EFS except that late deaths unrelated to lymphoma or treatment were not considered treatment failures | Randomization | Progression or relapse, initiation of a new anticancer treatment, death | Every 3 months for the first 2 years, every 6 months for the next 3 years | Thoracic and abdominal CT scans |
| MInT (Pfreundschuh, 2011) | Randomization | Progression, relapse, death; additional treatment was censored | Randomization | PD; non-CR/CRu; PR associated with treatment in excess of the protocol; no change; relapse; death | Every 3 months in the first 2 years, every 6 months thereafter | Physical examination, laboratory tests, chest and abdomen CT scans |
| ECOG4494/CALGB9793 (Habermann, 2006) | NA | NA | Randomization | Relapse, non-protocol treatment, or death | Every 3 months for the first 2 years, every 6 months for year 3, then annually | NA |
| RICOVER-60 (Pfreundschuh, 2008) | Randomization | Progression, relapse, death | Randomization | Progression, salvage treatment, additional (unplanned) treatments, relapse, death | Every 3 months for the first 2 years, every 6 months during the third to fifth year | Physical examination, laboratory tests, chest and abdomen CT scans |
| LNH03-2B (Récher, 2011) | Randomization | Progression, relapse, death | Randomization | Death, progression, relapse after CR/CRu, lymphoma treatment not stipulated by protocol | Every 3 months for the first 2 years, every 6 months thereafter | Physical examination, laboratory tests; chest, abdomen, pelvis CT scans |
| DSHNHL2002-1 (Schmitz, 2012) | Randomization | Progression, relapse, death | Randomization | Progression, start of salvage treatment, additional, unplanned treatment, relapse, or death | Every 3 months for the first 2 years, every 6 months for years 3 to 5, then annually | NA |
| ANZINTER3 (Merli, 2012) | NA | NA | Randomization | Non-CR, relapse, death, treatment interruption or change, late toxic events correlated to study treatment | NA | NA |
| LNH03-6B (Delarue, 2013) | Randomization | Progression, relapse, death | Randomization | Progression during or after (for PR) treatment, relapse for CR/CRu, death, introduction of a new treatment without evidence of progression or relapse (including radiotherapy) | Every 3 months in the first 2 years, every 6 months thereafter | Physical examination, laboratory tests, CT scans |
| NCT01793844 (Li, 2019) | Randomization | Progression, relapse, death | NA | NA | Every 3 months for 2 years, every 6 months for 3 years, then annually | Physical examination, laboratory tests, CT scans |
| UK NCRI (Cunningham, 2013) | Randomization | Progression, relapse, death | NA | NA | Every 3 months until 1 year, every 6 months until 2 years, then annually | Chest, abdomen, pelvis CT scans at 3 months and 12 months after treatment. PET scans if necessary |
| DLCL04 (Chiappella, 2017) | Randomization | Progression, relapse, death | Randomization | Progression, no response after 4 courses of chemotherapy, relapse, death | NA | Mainly CT scans |
| NCT00355199 (Cortelazzo, 2016) | Study entry | Progression, relapse, death | Study entry | Death, progression, or treatment discontinuation for any reason | NA | NA |
| Alliance/CALGB 50303 (Bartlett, 2019) | Randomization | Progression, relapse, death | NA | NA | NA | NA |
| FLYER (Poeschel, 2019) | Randomization | Progression, no change, unknown status at the end of study therapy, relapse after CR/CRu, death | Randomization | Progression, no change, unknown status at the end of study therapy, relapse after CR/CRu, death; additional treatment | Every 3 months for the first 2 years, every 6 months in years 3–5, then annually | Clinical examination, laboratory analysis, imaging techniques |
| NHL-001 (Xu, 2019) | Randomization | Progression, death | NA | NA | Every 3 months for the first year, every 6 months until 2 years, then annually | Neck, thorax, abdomen, and pelvis CT scans |
| AGMT-NHL13 (Jaeger, 2015) | Randomization | Progression, relapse, death | Randomization | Progression, relapse, death, initiation of new anticancer treatment, secondary malignancy, unacceptable toxicity | Every 4 months | CT scans; bone marrow biopsies if initial infiltrated |
| PRELUDE (Crump, 2016) | Study enrollment | Disease recurrence, death | NA | NA | Every 6 months until treatment discontinuation; | NA |
| REMARC (Thieblemont, 2017) | Randomization | Progression, relapse, death | NA | NA | During maintenance, every three cycles and at cycles six (6 months), 12 (12 months), and 21 (18 months), at the end of maintenance or time of discontinuation, then annually. | Contrast-enhanced CT scan; repeat PET in responding patients who were positive at randomization |
| PILLAR-2 (Witzig, 2018) | Randomization | Relapse, death | NA | NA | At 12-week intervals for the first 2 years, at 24-week intervals during years 3-4, then annually | NA |
| PETAL (Dührsen, 2018) | NA | NA | Randomization | Progression, relapse, change of therapy, toxicity-related discontinuation, death | NA | NA |
| LYSA/GOELAMS (Lamy, 2018) | NA | NA | Randomization | Progression, relapse, death | NA | NA |
| GOYA (Vitolo, 2017) | Randomization | Progression, relapse, death | NA | NA | NA | NA |
| MAIN (Seymour, 2014) | NA | NA | NA | NA | NA | NA |
| REMoDL-B (Davies, 2019) | Registration | Progression, death | NA | NA | Every 3 months for 1 year, every 6 months thereafter until 5 years’ total follow-up. | Physical examination, ECOG performance status assessment, routine laboratory tests and imaging |
| PHOENIX (Younes, 2019) | Randomization | Progression, relapse, death | Randomization | Progression, relapse, subsequent disease-specific therapy for PET positive or biopsy-proven residual disease after ≥6 cycles of R-CHOP, death | NA | NA |
| MabEase (Lugtenburg, 2017) | NA | NA | NA | NA | NA | NA |

*Death in the table is all-cause death.

Abbreviation: CR, complete response; CRu, unconfirmed complete response; CT, computed tomography; ECOG, Eastern Cooperative Oncology Group; EFS, event-free survival; NA, not available; PD, progressive disease; PET, positron emission tomography; PFS, progression-free survival; PR, partial response; R-CHOP, rituximab, cyclophosphamide, doxorubicin, vincristine and prednisone.
