# Supplementary material for: Association of progression-free or event-free survival with overall survival in diffuse large B-cell lymphoma after immunochemotherapy: a systematic review
Source: Leukemia. 2020 Jul 10;34(10):2576–91. doi: 10.1038/s41375-020-0963-1 (PMC7515849; doi:10.1038/s41375-020-0963-1)
Supplement: Supplementary file 5 — Supplemental Table 4 [file 41375_2020_963_MOESM5_ESM.docx]

**Supplemental Table 4.** The definition of progression-free survival in phase II trials and retrospective cohort studies.

| Study | PFS | |
| --- | --- | --- |
|  | Starting point | Event |
| **Phase II trial (n = 4)** | | |
| LNH2007-3B (Casasnovas, 2017) | Randomization | Progression, relapse, death or last follow-up |
| DENSE-R-CHOP-14 (Murawski, 2014) | Start of therapy | Progression, relapse or death |
| LNH2003-3 (Fitoussi, 2011) | Randomization | Progression, relapse, death or last follow-up |
| Niitsu, 2010 | Study entry | Progression, relapse, death or last follow-up |
| **Retrospective study (n = 47)** | | |
| Go, 2019 | Start of therapy | Progression, death or last follow-up |
| Lee, 2019 | Diagnosis | Progression, relapse or death |
| Morrison, 2019 | Start of therapy | Progression or death |
| Yim, 2019 | Diagnosis | Progression, death or last follow-up |
| Chen, 2018 | Diagnosis | Progression or death |
| Hosoda, 2018 | Start of therapy | Progression, relapse or death |
| Kim, 2018 | Start of therapy | Progression, death or last follow-up |
| Li, 2018 | Diagnosis | Progression, relapse, or death |
| Li, 2018 | Diagnosis | Progressive or death |
| Matsumoto, 2018 | Start of therapy | Progression, death or last follow-up |
| Sun, 2018 | Diagnosis | Progression, death or last follow-up |
| Go, 2017 | Start of therapy | Progression, death or last follow-up |
| Kanemasa, 2017 | Diagnosis | Progression, relapse, death or last follow-up |
| Li, 2017 | Diagnosis | Progression or last follow-up |
| Liu, 2017 | Diagnosis | Progression, relapse or disease-related death |
| Park, 2017 | Diagnosis | Progression, relapse or death |
| Song, 2017 | Diagnosis | Progression |
| Tsuyama, 2017 | Diagnosis | Progression, relapse, death or last follow-up |
| Alinari, 2016 | Diagnosis | Progression, death or censoring patients without event at 60 months |
| Prochazka, 2016 | Diagnosis | Recurrence, death or censoring |
| Seo, 2016 | Diagnosis | Recurrence, progression or death |
| Dabaja, 2015 | Completion of treatment | Relapse |
| El-Galaly, 2015 | Diagnosis | Progression, relapse, death or last follow-up |
| Gong, 2015 | Diagnosis | Progression, relapse, death or last follow-up |
| Kumar, 2015 | Diagnosis | Progression, relapse or death |
| Melchardt, 2015 | NA | NA |
| Nakajima, 2015 | Start of therapy | Progression, relapse or death |
| Dabaja, 2014 | Diagnosis | Progression or death |
| Mian, 2014 | Start of therapy | Progression or death |
| Castillo, 2013 | Diagnosis | Progression, death or last follow-up. |
| Hasimoto, 2013 | Diagnosis | Progression, relapse, death or last follow-up. |
| Kojima, 2013 | Diagnosis | Recurrence, progression or death |
| Lu, 2013 | Diagnosis | Progression or death |
| Ozbalak, 2013 | NA | NA |
| Shi, 2013 | Diagnosis | Progression or death |
| Tomita, 2013 | Start of therapy | Progression, death or last follow-up |
| Castillo, 2012 | Diagnosis | Progression, death or last follow-up |
| Huang, 2012 | Diagnosis | Progression or death |
| Li, 2012 | Diagnosis | Progression, death or last follow-up |
| Li, 2012 | Start of therapy | Progression, relapse, death or the last follow-up |
| Lin, 2012 | Diagnosis | Progression or death |
| Tomita, 2012 | Start of therapy | Progression, death or last follow-up |
| Sehn, 2011 | Diagnosis | Progression, relapse or death |
| Bari, 2010 | Diagnosis | Progression, relapse, death or last follow-up, |
| Ennishi, 2010 | Start of therapy | Progression, relapse, or the end date of the study |
| Phan, 2010 | NA | NA |
| Scandurra, 2010 | NA | NA |

*Death in the table is all-cause death except one study (Liu, 2017)

Abbreviation: NA, not available; PFS, progression-free survival.
