# Supplementary material for: Association of progression-free or event-free survival with overall survival in diffuse large B-cell lymphoma after immunochemotherapy: a systematic review
Source: Leukemia. 2020 Jul 10;34(10):2576–91. doi: 10.1038/s41375-020-0963-1 (PMC7515849; doi:10.1038/s41375-020-0963-1)
Supplement: Supplementary file 6 — Supplemental Figure and Table legends [file 41375_2020_963_MOESM6_ESM.docx]

**Supplemental figure legends**

**Supplemental Figure 1. Sensitivity Analysis by Leaving One Subgroup of Trials Out.** Sensitivity analysis in trial-level correlation of (A) PFS and (B) EFS, in treatment arm-level correlation of (C) 1-year PFS and 5-year OS, (D) 2-year PFS and 5-year OS, (E) 3-year PFS and 5-year OS, (F) 5-year PFS and 5-year OS, (G) 1-year EFS and 5-year OS, (H) 2-year EFS and 5-year OS, (I) 3-year EFS and 5-year OS and (J) 5-year EFS and 5-year OS. Correlation coefficient *r* was expressed as the value and its 95% confidence interval. Green circle and blue square represent the correlation in PFS and EFS, respectively. PFS, progression-free survival; EFS, event-free survival; OS, overall survival.

**Supplemental table legends**

**Supplemental Table 1.** **The assessment of risk of bias in randomized controlled trials by using the Cochrane Collaboration tool.**

Abbreviation: CHOP, cyclophosphamide, doxorubicin, vincristine, and prednisone; ECOG, Eastern Cooperative Oncology Group; G-CHOP, obinutuzumab, cyclophosphamide, doxorubicin, vincristine and prednisone; IPI, International Prognostic Index; ITT, intent-to-treat; IV, intravenous; PET, positron emission tomography; R, rituximab; R-ACVBP, rituximab, doxorubicin, cyclophosphamide, vindesine, bleomycin, and prednisone; R-CEOP70, rituximab, cyclophosphamide, epirubicin (70 mg/m2), vincristine, and prednisone; R-CEOP90, rituximab, cyclophosphamide, epirubicin (90 mg/m2), vincristine, and prednisone; R-CHOP, rituximab, cyclophosphamide, doxorubicin, vincristine and prednisone; R-CHOP50, rituximab, cyclophosphamide, doxorubicin (50 mg/m2), vincristine, and prednisone; R-CHOP-14, R-CHOP every 14 days; R-CHOP-21, R-CHOP every 21 days; R-miniCEOP, rituximab, cyclophosphamide, epirubicin, vinblastine, and prednisone; RA-CHOP, R-CHOP with bevacizumab; RB-CHOP, R-CHOP with bortezomib; SC, subcutaneous

**Supplemental Table 2. The assessment of risk of bias in non-randomized trials and retrospective studies by using the Newcastle – Ottawa quality assessment scale for cohort study.**

**Supplemental Table 3. The definition of progression-free survival and event-free survival and follow-up strategy in phase III randomized controlled trials.**

*Death in the table is all-cause death.

Abbreviation: CR, complete response; CRu, unconfirmed complete response; CT, computed tomography; ECOG, Eastern Cooperative Oncology Group; EFS, event-free survival; NA, not available; PD, progressive disease; PET, positron emission tomography; PFS, progression-free survival; PR, partial response; R-CHOP, rituximab, cyclophosphamide, doxorubicin, vincristine and prednisone.

**Supplemental Table 4. The definition of progression-free survival in phase II trials and retrospective cohort studies.**

*Death in the table is all-cause death except one study (Liu, 2017)

Abbreviation: NA, not available; PFS, progression-free survival.
